# Novichok Nerve Agents as Inhibitors of Acetylcholinesterase—In Silico Study of Their Non-Covalent Binding Affinity

**DOI:** 10.3390/molecules29020338

**Published:** 2024-01-09

**Authors:** Rafal Madaj, Bartłomiej Gostyński, Arkadiusz Chworos, Marek Cypryk

**Affiliations:** 1 Centre of Molecular and Macromolecular Studies, Polish Academy of Sciences, Sienkiewicza 112, 90-363 Lodz, Poland; r.madaj@uw.edu.pl (R.M.); arkadiusz.chworos@cbmm.lodz.pl (A.C.); 2 Institute of Evolutionary Biology, Faculty of Biology, Biological and Chemical Research Centre, University of Warsaw, Żwirki i Wigury 101, 02-089 Warsaw, Poland

**Keywords:** Novichok, AChE, DFT, binding affinity

## Abstract

In silico studies were performed to assess the binding affinity of selected organophosphorus compounds toward the acetylcholinesterase enzyme (AChE). Quantum mechanical calculations, molecular docking, and molecular dynamics (MD) with molecular mechanics Generalized–Born surface area (MM/GBSA) were applied to assess quantitatively differences between the binding energies of acetylcholine (ACh; the natural agonist of AChE) and neurotoxic, synthetic correlatives (so-called “Novichoks”, and selected compounds from the G- and V-series). Several additional quantitative descriptors like root-mean-square fluctuation (RMSF) and the solvent accessible surface area (SASA) were briefly discussed to give—to the best of our knowledge—the first quantitative in silico description of AChE—Novichok non-covalent binding process and thus facilitate the search for an efficient and effective treatment for Novichok intoxication and in a broader sense—intoxication with other warfare nerve agents as well.

## 1. Introduction

Synthetic organophosphorus inhibitors of acetylcholinesterase (AChE) are ubiquitous in many areas of human activity from agriculture: in the form of insecticides [1,2] through medicine: anti-inflammatory drugs and potential treatments of neurological diseases [3,4] to modern warfare: nerve agents [5]. Despite their potential medical use however, the majority of such applications are related to termination rather than sustaining life: the acute toxicity and lethality of these compounds is caused by their ability to irreversible impair the enzymatic ability to operate.

Due to its high turnover number, AChE is known to quickly hydrolyze the cholinergic neurotransmitter, acetylcholine (ACh) to acetic acid and choline [6]. By doing so, it reduces ACh concentration in the synaptic cleft and, therefore, terminates neural signalling. Any permanent inhibition of the enzyme would obviously induce an over-accumulation of ACh in the cholinergic system, the *cholinergic crisis* [7], causing overstimulation, a subsequent irreparable detriment of nerve cells and a fatal muscular paralysis, leading in consequence to prompt death [8].

**Figure 1:**
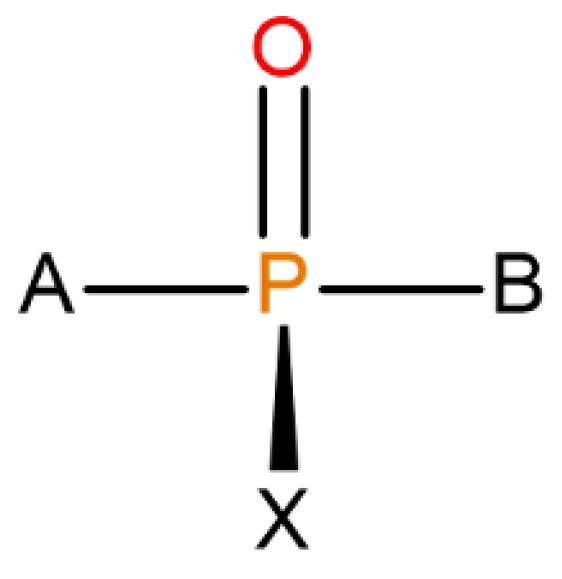
The overall structure of OPNAs’ (including Novichoks) organophosphorus core: the phosphoryl bond with X—halogen or another electron-withdrawing group (usually fluorine) and other organic or heteroorganic substituents (A,B).

**Scheme 1:**
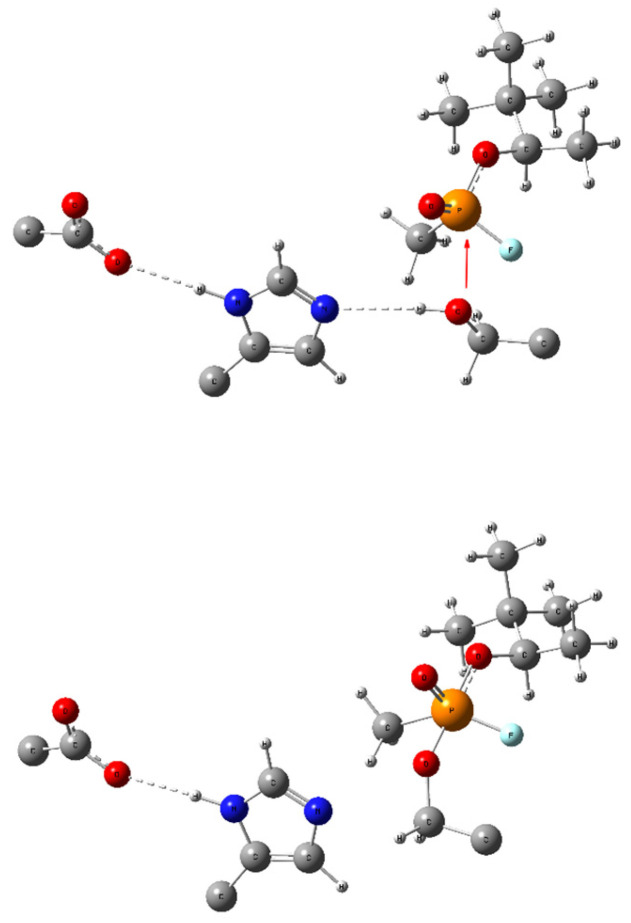
Bonding changes in the catalytic triad (GLU-HIS-SER, respectively) during the formation of a covalent bond between serine and an OPNA (here—soman). The red arrow symbolizes the nucleophilic attack of the Ser lone pair in the non-covalently bonded complex (up) leading to a pentacoordinate intermediate Ser-OPNA (low) and dashed lines symbolize hydrogen bonding. All redundant parts of the aminoacid scaffold were removed for clarity.

Organophosphorous compounds, especially the purpose-made, military-grade organophosphorus nerve agents (OPNAs) whose structures were assigned to halogen-containing phosphonate derivatives with their fluorinated organophosphorus core open to several modifications (see Figure 1), are believed to be able to precisely inhibit AChE by binding with one of the amino acids (serine) in the AChE catalytic triad (Ser-His-Glu) via a reactive hydroxyl group. A nucleophilic attack of Ser-OH towards the electrophilic phosphorus atom of a nerve agent in an AChE–OPNA non-covalent complex (Scheme 1) typically leads to the formation of a covalent bond between the serine and a given nerve agent (Ser-OPNA), therefore effectively impeding AChE functioning.

Numerous publications on various OPNAs’ working mechanisms have been published, e.g., [9,10] including ones that apply quantum mechanical (QM) methods [8,11] on the other hand—an efficient reactivation mechanism that could lead to a broad spectrum of possible antidotes has been explored at some depth only recently^3^ and the topic remains yet to be expanded.

The newest, A-generation of OPNAs, so-called “Novichoks” [12,13] which were developed shortly before the implementation of the Chemical Weapons Convention (CWC) in 1993, became anew a scientific point of interest after the infamous attempt of political poisoning of Sergei and Yulia Skripal in 2018 [10]. This renewal in scientific research on Novichoks comes arguably from both the fact that their structure and properties remain to a certain degree unknown and/or lacking particulars (as the Russian government never acknowledged developing them [11]) and the utmost threat to the public health and safety policies they should pose due to their alleged extreme toxicity (estimations of <0.1 mg lethal dose [12]).

**Figure 2:**
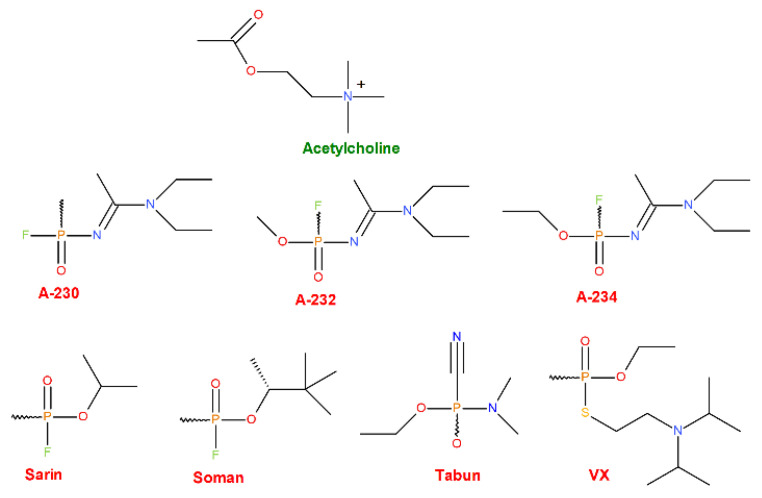
Structures of the AChE natural agonist (the upper-most row, green) and various synthetic OPNAs (middle and bottom rows, red) were chosen as ligands for our research.

The Novichoks’ most significant feature, distinguishing them from other, *classical* nerve agents, is the phosphonoamide bond, that replaced oxo- (sarin, soman, tabun) and tio- (VX) bonds between the phosphorus and heteroalkyl residues A or B (see Figure 1 and Figure 2) [14]. There are reports on the analysis of these compounds using theoretical and spectroscopic methods [9,10,13,15,16], and scarce in vitro research [17], yet there is, to the best of our knowledge, no information about any extensive in silico examinations of Novichoks’ mechanism of action and their potential reactivators. In reactivating the enzyme from OPNAs, several oxime-based compounds [18,19,20,21,22] as well as several engineered OPNA-degrading enzymes [14] are effective, and purely theoretical studies were conducted (e.g., [23,24]). Regarding Novichoks however, there is a lack of both QM and molecular mechanics (MM) investigations of Novichok–AChE interactions, including the noncovalent ones; yet, obviously, the noncovalent interplay between the ligand and the enzyme contributes to and facilitates the irreversible phosphonylation of the AChE catalytic-triad serine e.g., by adjusting the ligand electron density distribution therefore stabilizing the ligand in the enzyme active centre gorge and making it thus more susceptible to nucleophilic enzyme attack.

This work aims at in-depth investigating the human AChE–OPNAs binding affinities in comparison with the natural agonist as a starting point for further in silico research. Qualitatively and quantitatively assessed were the binding strengths of various OPNAs: the presently known, yet relatively unexplored A-series (A-230, A-232, A-234; “Novichoks”) and several arbitrarily selected, arguably the most publicly known and recognizable, examples of older OPNAs from the G- (tabun, sarin, soman) or the V-series (VX) for further comparison.

This unique assessment of the AChE binding affinity of the (officially) newest form of chemical weapon of mass destruction is in our opinion of at least twofold importance:

(a) From a purely academic point of view—it allows us to assess the strength of the interaction between the AChE enzyme and the A-tier OPNAs representatives for the first time. Thereby, the amount of scientific knowledge has increased, and new ideas will certainly emerge as to how to use this knowledge for the benefit of persons affected by those compounds. This particular aspect is strictly related to the next one;
(b) From the point of view of public health care and crisis management—this knowledge will contribute to the facilitation of counteracting the deadly effects of these weapons e.g., proposing new, more effective antidotes or as well as more effective pathways of removal of these substances from the human system or even blocking them from entering it in the first place.

## 2. Results and Discussion

Structures of the ligands selected for the research are depicted in Figure 2. whereas their average docking (${∆G}_{dock}$) and MM/GBSA binding energies (${∆G}_{bin}$) are included in Table 1. Coordinates for all G16-optimized ligand’s structures are available in Supplementary Materials.

The molecular docking provides a score that mainly indicates the overall thermodynamic value ${∆G}_{dock}$ for the docking (Table 1) and is regarded to be approximate [25] and always requires an indispensable refinement by an alternative method, in our case, by MM/GBSA method.

Presented data show that all the ligands are thermodynamically favourable in binding to the AChE active side. Interestingly, ACh is thermodynamically the least potent AChE ligand—as expected from a natural agonist of a high turnover number enzyme—with considerable discrepancies between the modest ACh binding energy value and the values calculated for the OPNAs. Taking into consideration the fact that all the selected OPNAs are warfare agents, designed especially to be irreversible AChE inhibitors—such results were expected and well prove the confirmation of the Novichoks’ and other OPNAs’ binding efficacy as compared to the ACh, at the first stages of the inhibition process, regardless of its possible mechanism.

The noteworthy fact is that the theoretical binding affinity for the newest (and implicitly: the most dangerous) Novichok tier is somewhat similar to the previous generation of warfare agents—at least for the first stage of the inhibition. Thus, considering $∆G_{bin}$ as one of the first components making up the ligand potency and toxicity, this is in agreement with the reports of Carlsen [16] whose QSAR model-based study disproves the existing claims as if the Novichoks were allegedly several times more potent than the hitherto known OPNAs.

The relatively high values of MM/GBSA standard deviations may be seen as a potential source of uncertainty regarding the correctness of the simulation results or protocols but this issue arises rather due to the method itself. MM/GBSA, although overall reliable and popular due to its moderate computational cost, is known to have incorporated several approximations with the method [26] that arguably are responsible for the somewhat diminished precision of outcomes.

**Figure 3:**
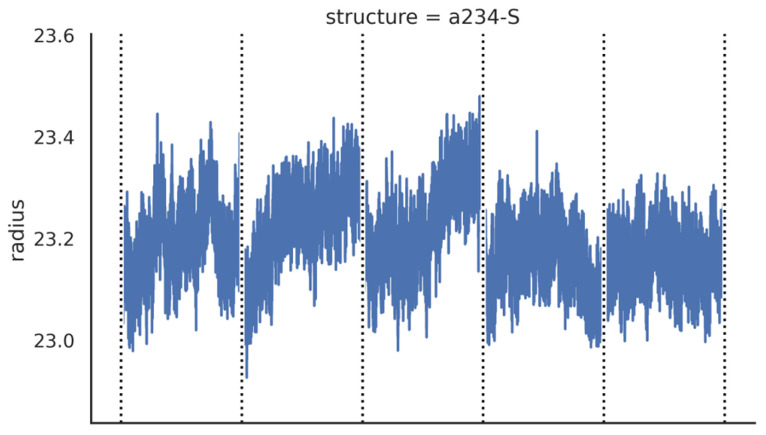
An exemplary plot presenting Rg = Rg(t) dependence for a selected Novichok. Plots for all simulated systems are available free of charge as Supplementary Materials (Supplementary Figures S1–S8).

Additionally, the spatial conformation and stability of the simulated AChE–ligand system are supported by stable values of the radius of gyration (R_g_) in Table 1 and R_g_ = R_g_(t) mapping trajectory plot in Figure 3. R_g_ that is practically constant over the entire molecular simulation process proves that no significant conformational change is initially induced in the AChE structure by the presence of the ligands.

Similar ligands and complex stabilities are supported by the root-mean-square deviation per residue (RMSD) and one of the AChE C_α_ atoms (Figures S9–S16 and S17–S24, respectively). The C_α_ RMSD is in the relatively low range of values. Such results are not supportive of the induced-fit binding and support the hypothesis that Novichoks’ (and other examined OPNAs’) binding mode engages conformations resulting from preexisting conformational dynamics; the secondary mode for the AChE enzyme was advocated e.g., by Xu et al. [27]

**Figure 4:**
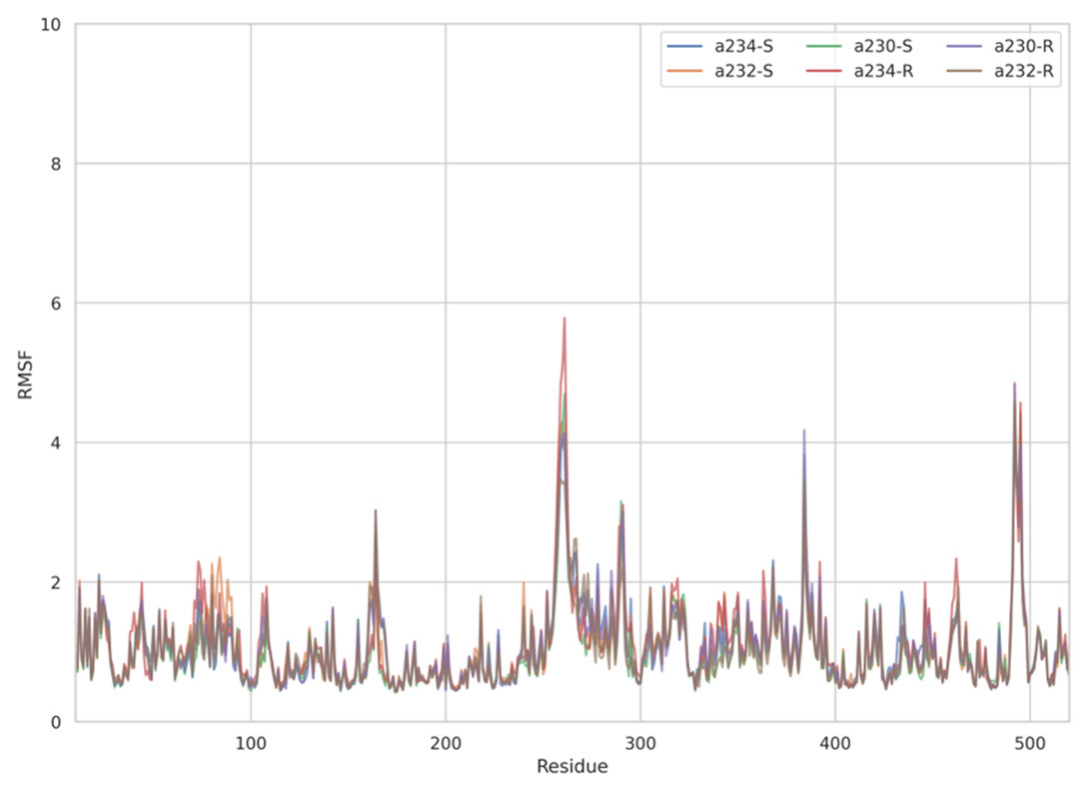
Plots of RMSF per residue [in Å] for the AChE–Novichok systems.

**Figure 5:**
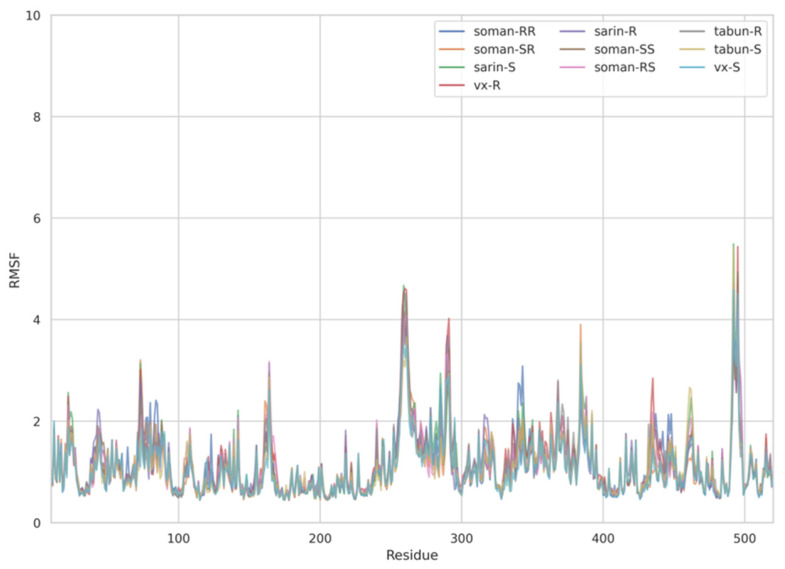
Plots of RMSF per residue [in Å] for the AChE—exemplary OPNAs systems.

**Figure 6:**
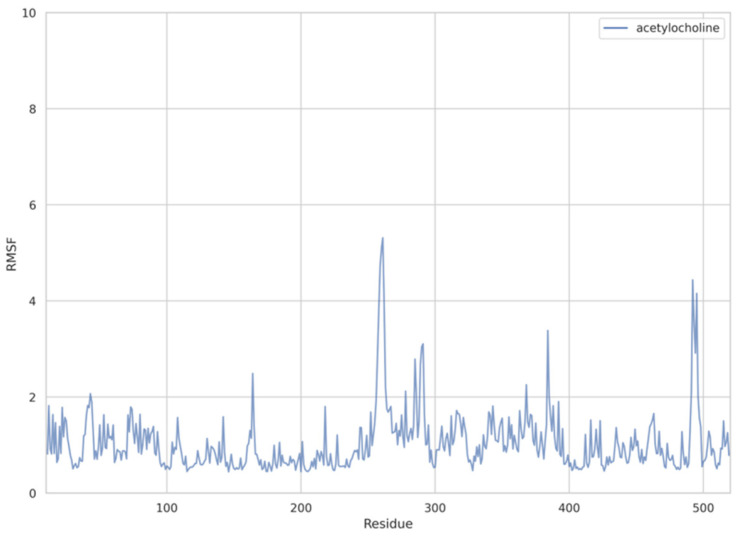
Plots of RMSF per residue [in Å] for the AChE—ACh system.

The dynamic adjustment similarity between the AChE—ACh and the AChE—OPNA systems is confirmed by RMSF calculations (Figure 4, Figure 5 and Figure 6). As can be seen, the root-mean-square fluctuations for examined systems are calculated for each residue separately (Figure 4, Figure 5 and Figure 6). The RMSF values were calculated disregarding the final five amino acids from both C- and N-termini to get rid of unnecessary fluctuations. Structural adjustments in the protein form a very similar pattern of spikes in each case and are furthermore of very similar magnitude. This allows us to presume that the protein structural folding adjusts to interact with Novichoks in the same way as with ACh and other OPNAs. This indicates that the putative Novichok working mechanism could be at least similar (if not identical) to the actual AChE—ACh mechanism.

The discrepancy between low RMSD values and higher RMSF most probably is due to the fact that RMSD values represent a quantitative measure of a structure divergence over time from the reference point, in this case, structures resulted from the energy minimization and the RSMF ones reveal which residues are the most mobile (fluctuate the most, i.e., diverge from the time-averaged structure).

**Figure 7:**
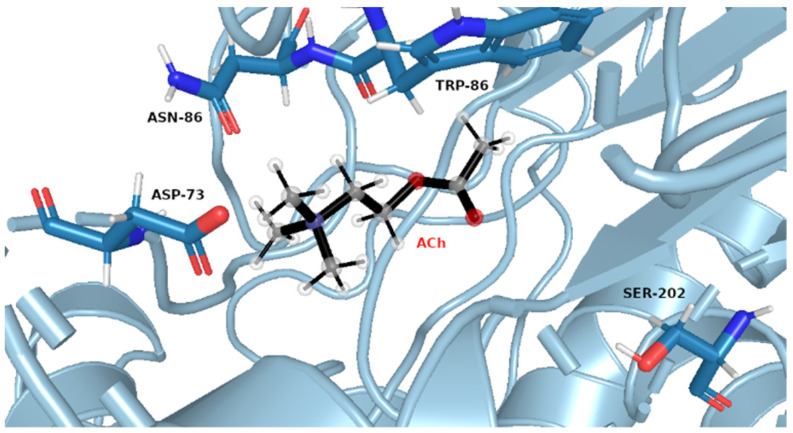
Exemplary snapshot of the most frequent structure for AChE complexes with ACh. Ser^202^ is shown for purely illustrative purposes.

**Figure 8:**
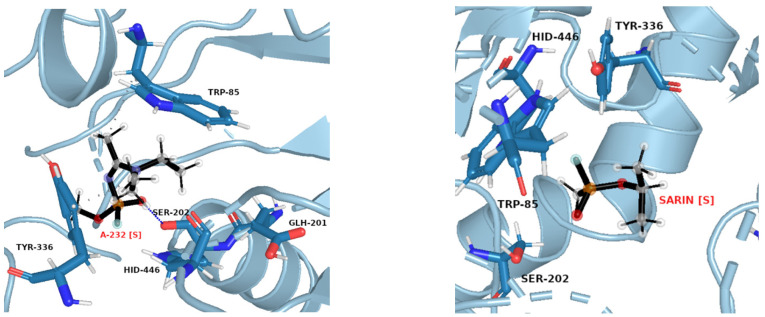
Exemplary snapshots of the most frequent structures for AChE complexes with A-232 [S] (**left**) and sarin [S] (**right**). The dashed line symbolizes hydrogen bonding.

The same pattern of flexibility and rigidity emerges when considering the SASA surface for different ligands (Figures S25–S32)—molecular volume enclosed by SASA remains relatively stable and of low values for each system, except for acetylcholine. The highest SASA average value and the highest SASA standard deviation values for ACh support the view of OPNAs buried deeper into the active site and being less flexible than ACh. This native ligand may in turn operate more freely and recruit for the active site solvent molecules. Such dynamics correspond well with the AChE high turnover number concerning its natural agonist. The most frequent structures from MD simulations (see Figure 7 and Figure 8), shown together with their corresponding interacting amino acids, support the image in which Novichoks and other OPNAs are placed much more closely to Ser202 than ACh.

**Figure 9:**
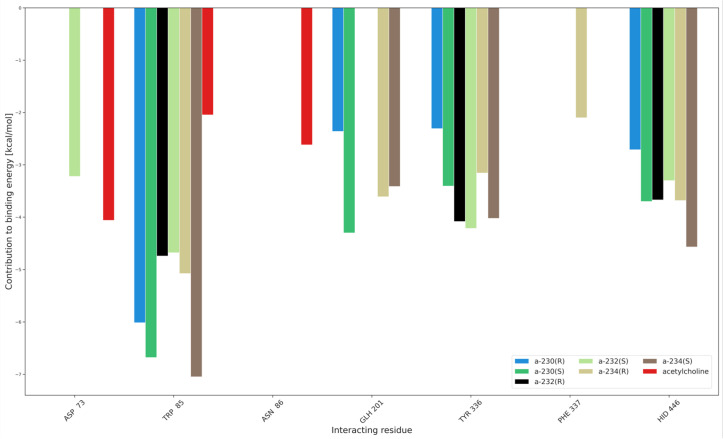
Interaction partition analysis for AChE complexes with Novichoks and ACh.

Additionally, the interaction partition analysis was performed in order to gain quantitative knowledge about how the interactions are divided between individual residues, i.e., with which amino acids the ligands interact the most. Exemplary partitioning for Novichoks is depicted in Figure 9 while Figures S33–S38 depict the amino acids and the interaction energy for the acethylcholine, Novichoks, and the remaining G- and V-series agents respectively as well as exemplary snapshots with all interacting residues common in the tier made visible.

As expected, our results show that the interactions within the active gorge of AChE are taking place between the ligands and amino acids that are placed in the nearest proximity of all the docked ligands. The main differences are the number of interacting residues (3 in the case of ACh, from 3 to 5 in the case of OPNAs) and their maximal strength in the tiers (about −4 kcal/mol for ACh, about −7 for A-234, slightly less than −7 for VX) and the particular residua themselves. Although there are amino acids that interact with most members of all the tiers (e.g., TRP 85), there are also ones that seem to be specific exclusively to OPNAs (TYR 336 or HID 446). This may be explained by the different sizes of the ligands and their different overall chemical construction. The results support all the previous considerations regarding the strength of ligand interactions and their flexibility.

The criteria of the final quality assessment were as follows: the best ligand was considered to be the one minimizing not only MM-GBSA energies but SASA outcomes as well because the low results of the latter reflect the ligand’s ability to remain in the binding site.

Considering all of the above factors, we conclude that the best binding OPNA turned out to be the S isomer of A-232, while the lowest affinity was shown by the RS diastereomer of soman. The worst ligand overall was acetylcholine, a fully expected outcome, considering the high turnover number of AChE.

## 3. Materials and Methods

### 3.1. Methodology

The workflow for this research consisted of the following steps:

1. Preparation and optimization of the protein and all ligands, including their stereoisomers;
2. Docking of each ligand to the AChE binding cavity, yielding preliminary quantitative forecasts as to whether the binding is thermodynamically favourable;
3. Molecular dynamics and MM/GBSA simulations of any thermodynamically favourable dockings, giving insights into how ligands may stabilize in the active site and yield the binding affinity of their non-covalent complexes.

#### 3.1.1. Preparation of the Protein and Ligands

The protein structure, downloaded from the RCSB PDB database [28] (PDB ID: 4m0e [29]), was reconstructed using Modeller 10.3 software to fill missing residues, then after removing ligands, it was subjected to structure validation using SAVES 6.0 server [30], passing the vast majority of tests, with high overall quality factor. The protein was protonated accordingly to its optimum pH using the Poisson-Boltzmann method [31] and prepared for molecular docking with the AMBER force field by merging non-polar hydrogens and calculating partial charges on residues.

Ligand structures (see Figure 2) were created using GaussView 6.0 [32], based on structures deposited in the PubChem database (https://pubchem.ncbi.nlm.nih.gov/, accessed on 27 October 2023), and subsequently optimized using one of the DFT methods (B3LYP-GD3/cc-pvtz level of theory) as implemented in Gaussian16 software [33]. Optimization and vibrational analysis for each ligand were performed for the gas phase. Additionally, the Merz–Kolmann population analysis and RESP partial charge [34] derivation were performed with HF/6-31G* level of theory. The latter resulted in a series of .gesp files that were subsequently used for the generation of .mol2 molecular dynamics input and library files by Antechamber 20.0 software [35] from AmberTools20 [36].

#### 3.1.2. Molecular Docking

The protein was protonated, non-polar hydrogens were merged and calculation of partial charges on its residues was performed as mentioned above. The charges as well as the number of torsion angles were calculated using AutoDockTools 1.5.6 [37]. The flexible residues were selected based on a centroid of the active site and extracted to separate output files, also using AutoDockTools 1.5.6. The molecular docking of all selected ligands was performed using AutoDockVina 1.1.2 [38]. The docking grid was set on a centroid of the active site with flexible residues and a total volume of a maximum of 27,000 Å^3^.

Every docking yielded up to 20 ligand conformations and was repeated 4 times for each ligand. Then the ligands in the vicinity of Ser202 with the lowest energy and optimized spatial structure of flexible residues were merged with input protein structure using an in-house Python script. This procedure yielded initial energies of binding and spatial orientation of ligands inside the protein’s active centre and subsequently served as starting points for molecular dynamics simulations.

#### 3.1.3. Molecular Dynamics

The Antechamber software was applied for the conversion of the ligand .gesp files to the input .mol2 ones with RESP partial charges assigned. Topology and input coordinate files were generated using tLeap, a module of the Amber20 package, using ff14SB force field [39] for protein, GAFF [40] for ligand, and TIP3P water model [41] for the solvent. The system was placed in a truncated octahedral box, solvated, and neutralized with sodium or chloride ions, depending on its total charge. MD simulation was performed with the Amber20 pmemd module, with minimizations and heating performed using multiple CPU threads, and productions using GPU. The first 10 ns of each production simulation were treated as an equilibration phase and omitted in further analysis. The trajectory was saved every 5000 MD step. Validation of the simulation was accomplished using CPPTRAJ 5.1.0 software [42], through the calculation of the radius of gyration (R_g_) of the complex, root-mean-square deviation (RMSD) of the ligand atoms related to the structure after optimization, the Cα atoms of the protein, root-mean-square fluctuations (RMSF) of system residues, solvent accessible surface area (SASA) of the ligand. Additionally, an interaction energy partitioning (i.e., contributions from amino acids that interact with the given ligand the most) was calculated for every 10th trajectory frame and averaged. In order to obtain the most frequent orientation of ligands within the binding cavity, the trajectories were clustered using a hierarchical agglomeration algorithm. The relative binding energies of ligand to the enzyme: ${∆G}_{bin}$ were calculated based on the MM/GBSA algorithm [26]. This yields relative binding energies with much higher accuracy than the one obtained through molecular docking and also indicates the most optimal conformation of the ligand within the binding cavity prior to covalent binding to the enzyme.

## 4. Conclusions

We think that the threat the possible usage of these compounds poses for the safety of public health is so significant that the current state of knowledge on this topic is far from being sufficient in order to take countermeasures effectively and efficiently at large. However, the failed Yulia and Sergei Skripal assassination attempt is apparent evidence that adequate help, when applied promptly, is able to obstruct the disastrous, fatal impact these compounds were designed to cause.

Therefore, this paper aims to slowly begin filling the yawning chasm between what *is already* known and what *should be* known regarding the Novichoks–acetylcholinesterase enzyme (AChE) interactions because, due to their putatively higher affinity to AChE, combined with recalcitrance to degradation and potential resistance to oxime’s reactivation mechanism, Novichoks is a significant threat that requires investigation. Data obtained by our research allow us to draw several conclusions about the Novichok-AChE binding affinity and provide certain clues regarding their possible working mechanism.

The selected organophosphorus nerve agents (OPNAs) bind to AChE in a thermodynamically favourable way and the natural agonist, acetylcholine (ACh), turns out to be the least potent binder with a huge discrepancy between itself and the OPNAs, which is expected from a high turnover-number enzyme. The comparable binding affinities of all the nerve agents support rather the scarce literature reports, according to which Novichoks are similar in potency to the older OPNAs instead of being much superior in toxicity.

The AChE-Novichok complexes are temporarily and spatially stable entities as indicated by the radius of gyration, root-mean-square deviation (RMSD), and C_α_ (alpha carbon) RMSD values. The results do not support the induced-fit binding mode; exploiting conformations from preexisting conformational dynamics offers a better explanation of the observed features.

ACh is the most flexible as indicated by e.g., solvent-accessible surface area values as well as the interaction partition analysis. Almost identical patterns of the OPNAs RMSF spikes and their magnitude, as compared to the ACh ones, prove the dynamic adjustment similarity which in turn indicates that the working mechanism may resemble the actual AChE—ACh mechanism or be identical. Although, while focusing in this work solely on the non-covalent binding as the very first step of the Novichok inhibition process, we intentionally refrain from unambiguously choosing any specific putative mechanism, we think that the results presented above allow us to surmise that the process in question should be the nucleophilic substitution at the phosphorus centre, proceeding either via pure S_N_2 mechanism or via the substitution-elimination (S–E) scheme, exactly as in the case of the rest of examined OPNAs.

This hypothesis however is outside the scope of the present paper and could be answered with more degree of certainty by more elaborate studies, engaging more sophisticated levels of theory applied (preferably, pure quantum mechanical (QM) or hybrid QM/MM methods with one of the ab initio approaches) allowing to follow and probe the reaction coordinate and obtain activation energy barriers. At present, we are in the process of verifying it.

## Figures and Tables

**Table 1:** An average docking score and MM/GBSA-binding energies [both in kcal/mol] for the selected ligands. An average radius of gyration (Rg) [in Å] and SASA [in Å^2^] for the selected ligands.

Compound	${∆G}_{dock}$ (Average)	$∆G_{bin}$ (Average)	R_g_ (Average)	SASA (Average)
A-230 (R)	−5.8 ± 0.2	−27.7 ± 8.2	23.17 ± 0.07	41.23 ± 61.2
A-230 (S)	−5.8 ± 0.2	−27.5 ± 7.5	23.18 ± 0.07	30.16 ± 21.2
A-232 (R)	−6.0 ± 0.2	−35.3 ± 13.0	23.15 ± 0.07	32.75 ± 23.1
A-232 (S)	−6.0 ± 0.2	−35.3 ± 8.4	23.14 ± 0.06	29.11 ± 27.5
A-234 (R)	−6.4 ± 0.1	−24.8 ± 6.4	23.21 ± 0.07	33.39 ± 23.1
A-234 (S)	−6.4 ± 0.1	−25.5 ± 8.1	23.19 ± 0.08	23.80 ± 16.1
acetylcholine	−5.4 ± 0.4	−6.6 ± 5.1	23.24 ± 0.08	60.15 ± 137.3
sarin (R)	−5.1 ± 0.3	−28.6 ± 6.4	23.20 ± 0.07	48.29 ± 18.0
sarin (S)	−4.6 ± 0.2	−31.9 ± 9.0	23.23 ± 0.06	69.82 ± 37.0
soman (RR)	−5.8 ± 0.2	−25.5 ± 6.4	23.23 ± 0.06	42.15 ± 35.4
soman (RS)	−5.9 ± 0.2	−25.8 ± 8.0	23.25 ± 0.08	62.13 ± 35.7
soman (SR)	−5.9 ± 0.2	−27.9 ± 6.9	23.20 ± 0.08	46.02 ± 25.2
soman (SS)	−5.9 ± 0.2	−27.3 ± 6.0	23.23 ± 0.07	57.14 ± 33.9
tabun (R)	−5.5 ± 0.3	−30.0 ± 7.1	23.21 ± 0.08	38.34 ± 20.5
tabun (S)	−4.9 ± 0.2	−28.0 ± 8.3	23.23 ± 0.08	36.72 ± 26.0
VX (R)	−5.9 ± 0.2	−36.9 ± 6.5	23.25 ± 0.09	53.40 ± 37.7
VX (S)	−6.4 ± 0.2	−32.3 ± 7.6	23.17 ± 0.07	45.75 ± 27.9

## Data Availability

Data are contained within the article and Supplementary Materials.

## Supplementary Materials

The following supporting information can be downloaded at: https://www.mdpi.com/article/10.3390/molecules29020338/s1, 1. Cartesian coordinates for all DFT-optimized ligands; 2. Figures S1–S8: Radius of gyration of protein-ligand complex as a function of simulation steps; 3. Figures S9–S16: RMSD of protein-ligand complex as a function of simulation steps; 4. Figures S17–S24: Cα RMSD of protein-ligand complex as a function of simulation steps; 5. Figures S25–S32: SASA of protein-ligand complex as a function of simulation steps; 6. Figures S33–S38: Interaction partitioning for the given ligands with exemplary snapshots.
