# Supplementary material for: Novichok Nerve Agents as Inhibitors of Acetylcholinesterase—In Silico Study of Their Non-Covalent Binding Affinity
Source: Molecules. 2024 Jan 9;29(2):338. doi: 10.3390/molecules29020338 (PMC10819560; doi:10.3390/molecules29020338)
Supplement: Supplementary file 1 [file molecules-29-00338-s001.zip › molecules-2778252-supplementary.pdf]

Novichok Nerve Agents as Inhibitors of Acetylcholinester-ase—In Silico Study of their Non-Covalent Binding Affinity

Rafał Madaj [a,b*], Bartłomiej Gostyński [a*], Arkadiusz Chworos [a], Marek Cypryk [a]

[a] *Centre of Molecular and Macromolecular Studies, Polish Academy of Sciences, Sienkiewicza 112, 90-363 Lodz, Poland*

[b] *Institute of Evolutionary Biology, Faculty of Biology, Biological and Chemical Research Centre, University of Warsaw, Żwirki i Wigury 101, 02—089 Warsaw, Poland*

e-mail: r.madaj@uw.edu.pl, bartlomiej.gostynski@cbmm.lodz.pl, arkadiusz.chworos@cbmm.lodz.pl, marek.cypryk@cbmm.lodz.pl

SUPPLEMENTARY INFO

Cartesian coordinates for all optimized ligands

Acetylcholine

N	1.81472200	0.09965400	0.07096600
C	0.56813000	-0.54277800	0.66129000
C	2.70784800	0.51120000	1.21159300
C	2.55313200	-0.88183800	-0.79646600
C	1.46441900	1.33071700	-0.72918400
C	-0.39946300	-1.15386800	-0.36566000
C	-2.29576300	0.21794100	-0.05371700
C	-3.73266400	0.22975800	0.35527800
O	-1.74405600	-1.02497200	0.09307200
O	-1.65095100	1.14898600	-0.46822900
H	0.06181800	0.24015000	1.21664000
H	0.91629500	-1.29841100	1.36183600
H	2.16573100	1.20291700	1.84991100
H	2.99414000	-0.37159900	1.77618900
H	3.59201200	0.99586800	0.80728500
H	1.93902200	-1.14170400	-1.65248000
H	2.77663000	-1.77036400	-0.21202300
H	3.47570000	-0.42140100	-1.13825900
H	0.81529300	1.06110600	-1.55268700
H	2.38757300	1.76731700	-1.09950300
H	0.93840500	2.02766400	-0.08546000
H	-0.23523500	-2.22206800	-0.47356700
H	-0.30287900	-0.68076700	-1.34129500
H	-3.84695300	-0.20870300	1.34593400
H	-4.10633100	1.24820900	0.34154500
H	-4.30805800	-0.38470000	-0.33838300

A230 [R]

N	-0.54604600	0.24385600	0.13208200
N	1.71824600	0.12162000	-0.00857800
C	0.50530500	-0.46175200	-0.15699100
C	0.47427200	-1.88717100	-0.66262100
C	2.98381700	-0.52328600	-0.35563800
C	3.67234600	-1.17554900	0.84206300
C	1.80778400	1.50311500	0.47811900
C	1.64511900	2.52597600	-0.64239200
C	-2.90899200	0.73089100	1.41839600
O	-2.54615500	-1.59614800	-0.04021700
P	-2.13151400	-0.17783400	0.06779700
F	-2.63967100	0.66977200	-1.18908400
H	0.71227200	-1.90499200	-1.72823400
H	1.20036400	-2.51359100	-0.14731000
H	-0.51973600	-2.30028400	-0.52829400
H	3.63097100	0.24406300	-0.78401300
H	2.82188100	-1.25357800	-1.14356800
H	4.63030800	-1.60383700	0.54397400
H	3.85888500	-0.44678600	1.63120200
H	3.05631900	-1.97139700	1.26036200
H	2.77816000	1.61468300	0.96286700
H	1.03856200	1.64900100	1.23195600
H	2.40365800	2.38899300	-1.41491100
H	0.66151700	2.42554900	-1.09750300
H	1.74367600	3.53863500	-0.24890400
H	-2.63359400	1.78236300	1.37644900
H	-3.98927600	0.62323200	1.33806800
H	-2.57829800	0.30500600	2.36424500

A230 [S]

N	-0.54843200	0.24600800	0.09440800
N	1.71579600	0.13289000	-0.04859600
C	0.50325900	-0.44356000	-0.22825500
C	0.47891600	-1.84124400	-0.80756700
C	2.98440000	-0.50180600	-0.40299300
C	3.64765000	-1.21416300	0.77494400
C	1.80452200	1.47997100	0.52635900
C	1.66732000	2.57247000	-0.52989500
O	-2.57662200	-1.44704200	-0.57101100
P	-2.13383100	-0.17852600	0.05338100
H	0.83380600	-1.82439300	-1.83969500
H	1.12689200	-2.51206400	-0.24462900
H	-0.53489600	-2.22511300	-0.80185200
H	3.64327300	0.28086900	-0.78324900
H	2.83361300	-1.19466200	-1.22589700
H	4.60802900	-1.63444300	0.47327100
H	3.82487300	-0.52519900	1.60100300
H	3.01976500	-2.02470900	1.14426700
H	2.76735200	1.55579800	1.03244900

H	1.02319000	1.58223700	1.27480100
H	2.43695800	2.47909300	-1.29793300
H	0.69074600	2.50839600	-1.00652800
H	1.76494400	3.55782700	-0.07217000
C	-2.99615000	1.29152500	-0.54005900
H	-4.06859700	1.13602000	-0.43585600
H	-2.68868100	2.16739800	0.02694500
H	-2.75895300	1.43676900	-1.59278800
F	-2.51254600	-0.13567100	1.60437100

A232 [R]

N	-0.25176300	0.24003700	-0.03475800
N	2.01068900	0.12488300	0.04023400
C	0.82294100	-0.46450000	-0.22243300
C	0.84492900	-1.88952000	-0.72842500
C	2.04342800	1.50498900	0.54206300
C	1.98685700	2.53312900	-0.58367700
C	3.30729900	-0.51395000	-0.18068800
C	3.87707800	-1.16319300	1.07940400
O	-2.24564400	-1.55997300	-0.52805000
P	-1.80823300	-0.18011400	-0.22792200
H	1.21630700	-1.91177400	-1.75487000
H	1.49646500	-2.51821800	-0.12379900
H	-0.15978200	-2.29797600	-0.72106800
H	2.96077300	1.61728000	1.12065200
H	1.20178200	1.64158000	1.21587700
H	2.04495500	3.54322900	-0.17616800
H	2.81599000	2.40206200	-1.28114700
H	1.05070000	2.43526600	-1.13016800
H	3.98935800	0.25694000	-0.54331900
H	3.22721500	-1.24507200	-0.98037100
H	4.86168700	-1.58684000	0.87700300
H	3.98142800	-0.43390000	1.88294500
H	3.22671700	-1.96213200	1.43518600
F	-2.29012000	0.81278000	-1.37069400
O	-2.48845700	0.46934600	1.06516900
C	-3.87583200	0.19476100	1.32553000
H	-4.08804100	-0.86634300	1.19921800
H	-4.06141400	0.49474300	2.35367900
H	-4.50944600	0.77813000	0.65549000

A232 [S]

N	-0.23729500	0.14748300	0.24788500
N	2.00685400	0.18153200	-0.06501500
C	0.82376400	-0.45999200	-0.18982600
C	0.83298800	-1.82509900	-0.84089200
C	-3.92547100	0.74304500	-0.78602100
C	2.05221500	1.50880600	0.56298500
C	1.73560400	2.62836000	-0.42387600
C	3.28031900	-0.34889700	-0.54976500
C	4.08041100	-1.06797500	0.53506300
O	-2.21032100	-1.68835500	-0.19894900
O	-2.52405100	0.86798800	-0.48795500
P	-1.78443800	-0.34538900	0.24860700
F	-2.20900400	-0.10466200	1.75787300
H	1.05463500	-1.72715500	-1.90533000
H	1.59159900	-2.47021900	-0.40028100
H	-0.14113100	-2.28995800	-0.73635700
H	-4.16014100	1.52837000	-1.50000200
H	-4.51880800	0.88378800	0.11889900
H	-4.14096000	-0.23399900	-1.21732900
H	3.05117100	1.63464700	0.98120100
H	1.34018500	1.52168100	1.38399500
H	1.81221800	3.59866300	0.06849900
H	2.43013400	2.62072700	-1.26577600
H	0.72206600	2.51347000	-0.80356100
H	3.85388200	0.49400700	-0.93905200
H	3.10742400	-1.01084200	-1.39368900
H	5.03859200	-1.40715900	0.13916600
H	4.27810600	-0.40712800	1.37922600
H	3.53770800	-1.93574500	0.90955600

A234 [R]

N	-0.08272000	-0.04585600	0.37459500
N	-2.27440200	0.30710100	-0.08127400
C	-1.15522300	-0.43335800	-0.24605900
C	-1.24339000	-1.64249900	-1.15041500
C	3.73370300	0.29949700	-0.29186500
C	4.26529200	1.47753700	-1.07702000
C	-2.24231700	1.48956000	0.78960500
C	-1.72408600	2.72891500	0.06627500
C	-3.54539700	0.02822600	-0.74797100
C	-4.50896800	-0.78325700	0.11641500
O	1.71996000	-1.99280400	-0.27114800
P	1.40131400	-0.70722200	0.38581300
H	-1.35609900	-1.32255200	-2.18807200
H	-2.10045500	-2.26528600	-0.89863600
H	-0.33502400	-2.22934400	-1.07016900
H	4.23187300	0.21292200	0.67666100
H	3.86356200	-0.63865600	-0.83250500
H	5.33530700	1.35670400	-1.25240800

H	4.10619200	2.40736600	-0.53116200
H	3.76214600	1.55249800	-2.04089000
H	-1.61113800	1.26524200	1.64550200
H	-3.25749300	1.65154000	1.15275700
H	-1.74647000	3.59219800	0.73267300
H	-2.33546400	2.96056300	-0.80766400
H	-0.69693700	2.56856500	-0.25603300
H	-3.36405900	-0.47979000	-1.69102600
H	-3.99373600	0.98978900	-1.00377800
H	-5.45662200	-0.92860800	-0.40392400
H	-4.09255400	-1.76288600	0.35040800
H	-4.71468600	-0.27368000	1.05791000
F	1.71469500	-0.78737100	1.93886200
O	2.32152100	0.52051200	-0.06430700

A234 [S]

N	-0.09986500	-0.14879300	0.14192200
N	2.11863300	0.31846600	0.02810500
C	1.11663200	-0.59127400	0.04204800
C	1.49776000	-2.05201400	-0.04430400
C	-2.73696000	1.33341100	-0.29333800
C	-3.79525500	1.88101200	-1.22485900
C	1.80334500	1.74961500	0.10051300
C	1.59562200	2.23008900	1.53368200
C	3.53840800	-0.02938200	-0.01824900
C	4.11706100	0.00220400	-1.43170100
O	-1.58975300	-2.39152400	-0.25677700
O	-2.55203100	-0.06244300	-0.60985400
P	-1.50621700	-0.98307600	0.16179700
F	-1.96789300	-0.78008900	1.67137200
H	1.94184200	-2.37402900	0.89983000
H	2.22509400	-2.23105700	-0.83423800
H	0.61272100	-2.65095700	-0.22983000
H	-3.04299700	1.42207400	0.75102400
H	-1.78545100	1.85325200	-0.41743600
H	-3.96542300	2.93834300	-1.01595400
H	-4.73496000	1.34519000	-1.09493600
H	-3.48124100	1.77753000	-2.26325300
H	0.90864500	1.93201300	-0.48981300
H	2.62738400	2.28741800	-0.36892100
H	1.38958600	3.30126900	1.54828500
H	2.48325300	2.04899300	2.14199800
H	0.75263700	1.70865700	1.98308200
H	3.69338000	-1.00540800	0.43296600
H	4.06680100	0.68384300	0.61681200
H	5.18455600	-0.22115600	-1.41097600
H	3.62562700	-0.73109800	-2.07086500
H	3.98604100	0.98412900	-1.88696600

Sarin [R]

C	-1.51801400	1.44172300	0.75248500
C	1.54115200	0.08732900	0.20570000
C	2.21194200	-1.27241400	0.14150800
C	2.42435500	1.22610700	-0.26626100
O	-2.07866500	-0.45467600	-1.20268200
O	0.37508600	0.06150000	-0.67337000
P	-1.12604800	-0.06126300	-0.16256300
F	-0.98904700	-1.11672000	1.02405900
H	-0.76102400	1.65756700	1.50457700
H	-1.57801700	2.26709200	0.04562700
H	-2.48442000	1.31628200	1.23695800
H	1.20460800	0.27817800	1.22792200
H	3.09760800	-1.28557400	0.77811300
H	1.52994200	-2.05143700	0.47767200
H	2.51430000	-1.49126200	-0.88269300
H	1.89502900	2.17680700	-0.20967000
H	3.32037400	1.28958800	0.35197100
H	2.72576700	1.06111900	-1.30073400

Sarin [S]

C	-1.54117100	0.08730200	0.20571600
C	-2.42418800	1.22624300	-0.26620000
C	-2.21217900	-1.27233100	0.14143800
O	2.07867700	-0.45449900	-1.20274300
O	-0.37510400	0.06129200	-0.67333200
P	1.12604600	-0.06128700	-0.16256300
H	-1.20461300	0.27802400	1.22795500
H	-3.32017100	1.28988800	0.35206800
H	-1.89468600	2.17684600	-0.20962900
H	-2.72566900	1.06131600	-1.30066300
H	-1.53030500	-2.05148200	0.47756400
H	-3.09785600	-1.28538900	0.77803200
H	-2.51455900	-1.49107700	-0.88277800
F	0.98918500	-1.11680800	1.02400800
C	1.51796500	1.44167800	0.75254800
H	2.48446500	1.31626700	1.23682200
H	1.57777100	2.26712300	0.04578400
H	0.76111800	1.65735700	1.50481000

Soman [RR]

C	-0.71716600	0.65466300	-0.19948000
C	-0.92400100	2.11267500	0.17766600
C	-1.93763500	-0.28006900	-0.02272400
C	-1.53573100	-1.70573600	-0.43470000
C	-2.41949900	-0.29147200	1.43464500
C	-3.06832700	0.20040800	-0.94681900
O	0.36597500	0.14577700	0.63912000
O	2.60839500	-0.98994400	1.09778800
P	1.82969300	-0.20871000	0.13401600
H	-0.40299300	0.58711200	-1.24434100
H	-0.01036600	2.67836000	-0.00028400
H	-1.71715700	2.56324900	-0.41610800
H	-1.17609700	2.20726400	1.23202900
H	-0.76615400	-2.10727400	0.22176000
H	-2.40176100	-2.36651600	-0.38149900
H	-1.15370600	-1.73142400	-1.45656200
H	-3.22614500	-1.01620300	1.55241300
H	-1.61099900	-0.57167400	2.10873300
H	-2.80530800	0.68020800	1.74418500
H	-3.89840600	-0.50588900	-0.91168700
H	-3.45633500	1.17484300	-0.65087400
H	-2.73470400	0.26590900	-1.98495200
F	1.54470500	-0.97359100	-1.23375900
C	2.59350700	1.30433900	-0.47938800
H	2.75434000	1.97819200	0.35984500
H	3.55390300	1.05419000	-0.92602300
H	1.96232200	1.78710100	-1.22350300

Soman [RS]

C	0.72448400	0.55008900	-0.32087500
C	1.96395100	-0.29978200	0.04327200
C	2.40903300	-0.03984900	1.48930900
C	1.60927700	-1.78685100	-0.11775500
C	3.10656100	0.04118700	-0.92655400
O	-0.35369400	0.17021700	0.58935400
O	-2.79894100	-0.07360100	1.29719700
P	-1.86699800	-0.06862100	0.16727800
H	1.59434500	-0.22652200	2.18764300
H	3.23824600	-0.69999900	1.74739300
H	2.75091800	0.98561200	1.62888700
H	2.48062800	-2.40743200	0.09375200
H	0.81528100	-2.07970200	0.56703300
H	1.28482500	-2.00839900	-1.13705200
H	3.95034700	-0.62779500	-0.75379800
H	2.79707300	-0.07855700	-1.96739600
H	3.46518000	1.06124400	-0.79390800
C	0.88505200	2.05898500	-0.23285100
H	-0.04640900	2.54226000	-0.51995700
H	1.12743700	2.36284300	0.78363600

H	1.66912800	2.40781200	-0.90276200
H	0.42756500	0.28301500	-1.33963800
C	-1.93144400	-1.50275700	-0.92244100
H	-2.94170500	-1.58887600	-1.31791300
H	-1.22554800	-1.41091500	-1.74522600
H	-1.69835200	-2.39166800	-0.34010100
F	-2.11826600	1.11192500	-0.87386700

Soman [SR]

C	-0.72444900	0.55009800	-0.32079700
C	-0.88500200	2.05900000	-0.23269700
C	1.93124500	-1.50298900	-0.92219600
C	-1.96390900	-0.29976200	0.04337800
C	-1.60918700	-1.78683600	-0.11751100
C	-2.40906600	-0.03975300	1.48937900
C	-3.10645400	0.04110200	-0.92655200
O	0.35374500	0.17029000	0.58943400
O	2.79912600	-0.07321400	1.29692700
P	1.86699200	-0.06858900	0.16716800
F	2.11800600	1.11165000	-0.87439400
H	-0.42754900	0.28306400	-1.33957300
H	0.04642900	2.54226900	-0.51990400
H	-1.66917400	2.40788400	-0.90246400
H	-1.12722800	2.36279100	0.78384800
H	2.94143500	-1.58923400	-1.31781200
H	1.69818900	-2.39176300	-0.33964200
H	1.22521200	-1.41127300	-1.74488500
H	-0.81535900	-2.07964700	0.56749100
H	-2.48058500	-2.40741600	0.09380500
H	-1.28446800	-2.00842700	-1.13671100
H	-3.23809300	-0.70010200	1.74755300
H	-1.59432000	-0.22611900	2.18772900
H	-2.75122900	0.98562800	1.62883900
H	-3.95029300	-0.62779000	-0.75370500
H	-3.46500900	1.06121100	-0.79410600
H	-2.79692800	-0.07885600	-1.96735800

Soman [SS]

C	0.71715200	0.65464200	-0.19939400
C	-2.59330400	1.30435900	-0.47975500
C	1.93764100	-0.28006200	-0.02262600
C	2.41942600	-0.29153400	1.43476900
C	1.53580400	-1.70571800	-0.43470500
C	3.06836800	0.20050300	-0.94663200
O	-0.36600900	0.14569000	0.63914100
O	-2.60859300	-0.98973700	1.09769600
P	-1.82971100	-0.20869400	0.13391700
F	-1.54465200	-0.97375700	-1.23374100
H	-3.55368500	1.05424900	-0.92644400
H	-2.75414200	1.97834000	0.35937400

H	-1.96200400	1.78695400	-1.22388000
H	1.61090100	-0.57181200	2.10879600
H	3.22609600	-1.01623800	1.55253500
H	2.80517700	0.68014300	1.74439100
H	2.40184900	-2.36647500	-0.38148400
H	0.76619500	-2.10731300	0.22168100
H	1.15384600	-1.73136100	-1.45659400
H	3.89846000	-0.50578000	-0.91150600
H	2.73479600	0.26606800	-1.98477700
H	3.45634300	1.17492500	-0.65060100
C	0.92393100	2.11264500	0.17781900
H	0.01028200	2.67830800	-0.00012500
H	1.17599900	2.20719500	1.23219300
H	1.71708700	2.56327200	-0.41591500
H	0.40302500	0.58712100	-1.24427100

Tabun [R]

N	0.70391700	2.74772600	-0.96216900
N	1.00222300	-0.88086100	-0.43477200
C	0.59569400	1.77137900	-0.36099400
C	-1.99554500	0.03579200	-0.60826900
C	-3.39874500	-0.37213700	-0.22400100
C	2.39011400	-0.72720600	-0.87968300
C	0.61562500	-2.27757400	-0.20881400
O	0.94182600	0.40864000	1.96623600
O	-1.18773200	0.02121200	0.59940700
P	0.38599300	0.26157200	0.61392500
H	-1.97325200	1.04324800	-1.02704600
H	-1.55858300	-0.64840100	-1.33698500
H	-4.04080700	-0.35659100	-1.10549100
H	-3.40863300	-1.37800400	0.19487300
H	-3.80744400	0.31433800	0.51613300
H	2.61703600	0.31979100	-1.07187800
H	3.10151200	-1.10968800	-0.14150300
H	2.52169500	-1.27519600	-1.81345900
H	-0.41192300	-2.33500800	0.14221000
H	0.69189300	-2.82179700	-1.15124100
H	1.26002200	-2.76466100	0.52951900

Tabun [S]

N	-0.70292500	2.74788300	-0.96204800
N	-1.00255600	-0.88060400	-0.43484900
C	-0.59512100	1.77146200	-0.36091900
C	1.99558200	0.03539600	-0.60826400
C	3.39875200	-0.37262800	-0.22399800
C	-0.61647100	-2.27747600	-0.20896000
C	-2.39042000	-0.72643000	-0.87968000
O	-0.94168300	0.40873400	1.96626700
O	1.18773800	0.02066800	0.59938800
P	-0.38591600	0.26154200	0.61394500
H	1.55862800	-0.64871700	-1.33705900
H	1.97332400	1.04290400	-1.02692000
H	4.04084100	-0.35700700	-1.10546500
H	3.80744800	0.31374700	0.51622900
H	3.40857500	-1.37854400	0.19476300
H	0.41109500	-2.33530300	0.14194500
H	-1.26097500	-2.76432900	0.52943500
H	-0.69304800	-2.82164200	-1.15139100
H	-2.61699800	0.32066700	-1.07173700
H	-2.52224100	-1.27426900	-1.81350800
H	-3.10190700	-1.10875000	-0.14150300

VX [R]

N	-2.39880200	0.15433700	-0.28128400
C	-1.34528600	-0.79766700	0.01153800
C	-3.78170000	-0.28253000	-0.06180400
C	-4.22724700	-1.21546800	-1.19281100
C	-4.06537600	-0.92542600	1.30866100
C	-2.04557500	1.54867100	0.03133600
C	-2.68741100	2.51992900	-0.96121800
C	-2.32337200	1.97283100	1.48166700
C	-0.34602000	-0.84838500	-1.14686100
O	1.51127100	0.96405100	0.41153000
P	2.17053900	-0.36984000	0.39902500
S	1.17610000	-1.82929700	-0.76810500
H	-1.76109000	-1.79393200	0.15251600
H	-0.81339400	-0.55025500	0.93846500
H	-4.39356300	0.61785300	-0.12705800
H	-3.63250000	-2.13158100	-1.19923000
H	-5.27339900	-1.50446600	-1.07527600
H	-4.10273100	-0.72670400	-2.15828400
H	-5.13486400	-1.11203200	1.42153900
H	-3.74559400	-0.28578600	2.12840800
H	-3.55748000	-1.88560200	1.41218300
H	-0.96527600	1.61073000	-0.10731200
H	-2.32524200	3.53460800	-0.78853600
H	-3.77459600	2.54173900	-0.86375900
H	-2.44520700	2.23198100	-1.98390600
H	-1.91308400	2.96629300	1.66870600

H	-1.85815200	1.28466500	2.18813200
H	-3.39412500	2.01050100	1.68978400
H	-0.03013500	0.14944900	-1.43384500
H	-0.78720400	-1.33422000	-2.01558300
O	3.64923300	-0.40885300	-0.24532200
C	4.56847900	0.68424100	0.01303500
H	5.55723200	0.22731000	0.02273700
H	4.37782600	1.10743400	1.00221700
C	4.45229000	1.74854200	-1.05882100
H	4.62320100	1.31360100	-2.04330300
H	3.45949200	2.19435500	-1.03712400
H	5.19538600	2.53021400	-0.88980900
C	2.38994900	-1.08086600	2.05555900
H	3.03994100	-0.42573700	2.63609200
H	2.83511100	-2.07135200	1.98272500
H	1.41896500	-1.14788000	2.54148100

VX [S]

N	2.17806900	-0.28921900	-0.18906400
C	0.93394900	0.29411100	-0.65083000
C	3.40372400	0.48788500	-0.40573200
C	3.83975100	0.38706600	-1.87114200
C	3.34352000	1.96204700	0.03678900
C	2.04424800	-1.05037200	1.06416400
C	3.00944900	-2.23637700	1.10066500
C	2.15360100	-0.20697100	2.34414500
C	0.11481800	-0.74027500	-1.42666300
C	-4.03902100	-1.49153300	-0.31483100
C	-2.30318900	1.71657900	1.35412100
C	-1.96830700	2.90278900	0.47289200
O	-1.56412500	-1.29945000	1.00188100
O	-3.13315100	0.76469600	0.64245500
P	-2.51052200	-0.61271700	0.08345200
S	-1.60354700	-0.17155700	-1.79664500
H	1.13198300	1.13997200	-1.30721100
H	0.33056600	0.68309000	0.17775600
H	4.17493800	0.00367800	0.19423400
H	3.09299100	0.83367000	-2.53144100
H	4.78185100	0.91262400	-2.03846600
H	3.96203000	-0.65634400	-2.15885400
H	4.32824100	2.42286100	-0.05931000
H	3.02445300	2.06060400	1.07209700
H	2.65575600	2.53794700	-0.58444200
H	1.03235100	-1.45737000	1.04764900
H	2.81591600	-2.85641300	1.97732500
H	4.05068200	-1.91265800	1.15483300
H	2.89133800	-2.84876500	0.20712200
H	1.90199100	-0.81187700	3.21642800
H	1.46495100	0.63843000	2.31850600
H	3.16523100	0.17735500	2.48578800

H	0.04121000	-1.67132200	-0.87415000
H	0.56973700	-0.94420500	-2.39463300
H	-4.63543500	-0.90624600	-1.01233000
H	-3.78234600	-2.44883000	-0.76233800
H	-4.59686200	-1.64680600	0.60753700
H	-2.88926100	2.02028800	2.22082100
H	-1.40762000	1.21040900	1.71682200
H	-1.39268000	3.63459600	1.04254100
H	-2.87938400	3.38195000	0.11500800
H	-1.38158300	2.59190000	-0.38946700

Figures S1-S8

Radius of gyration of protein-ligand complex as a function of simulation steps

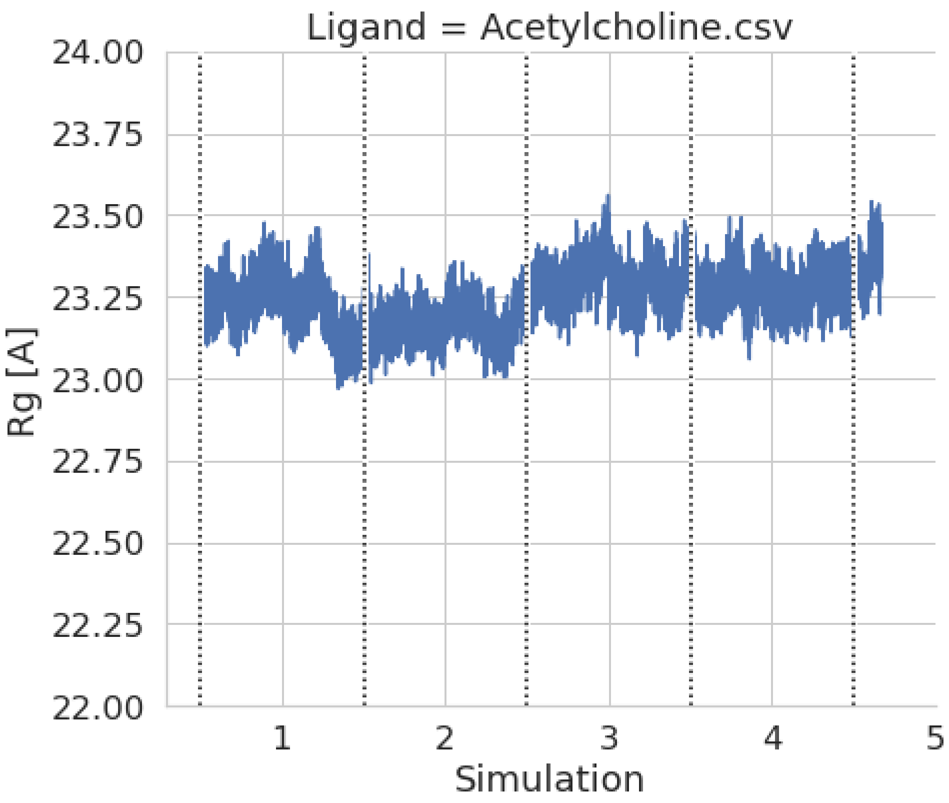

Figure S1. Radius of gyration of protein-acetylcholine complex as a function of simulation steps

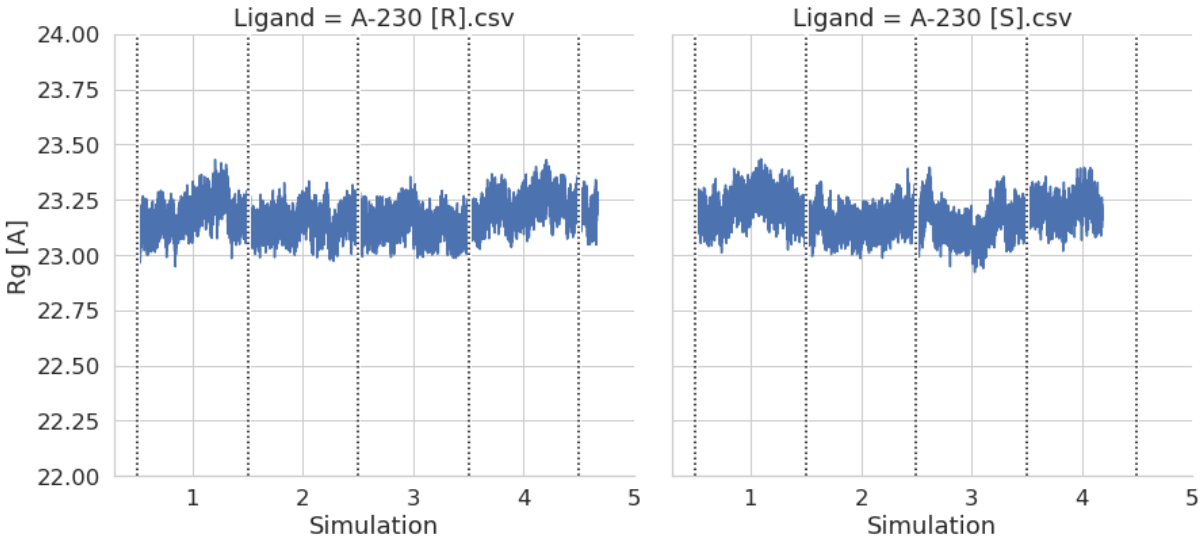

Figure S2. Radius of gyration of protein-A-230 complex as a function of simulation steps

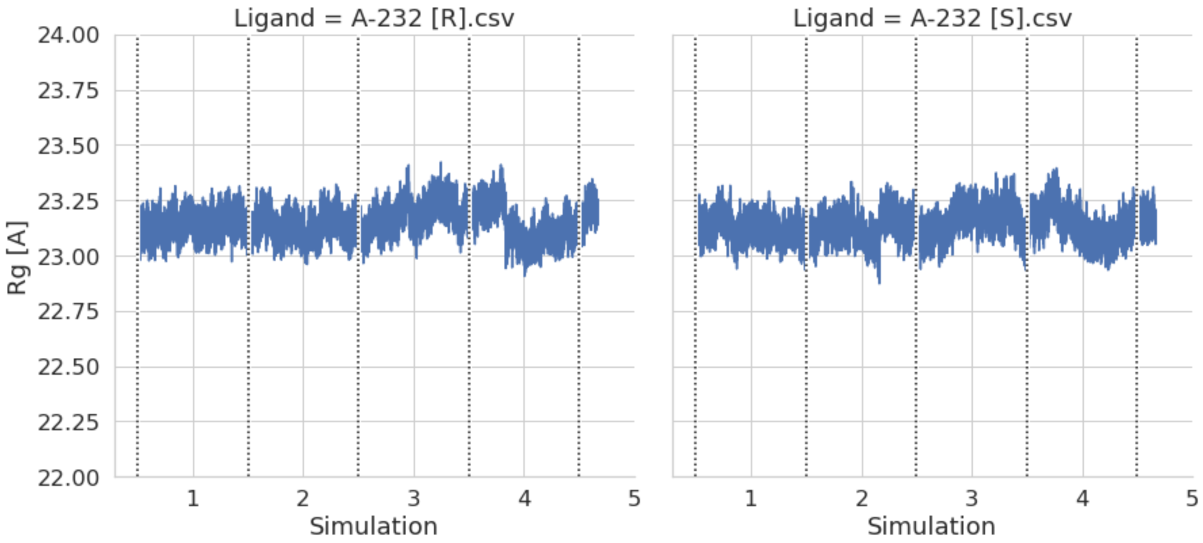

Figure S3. Radius of gyration of protein-A-232 complex as a function of simulation steps

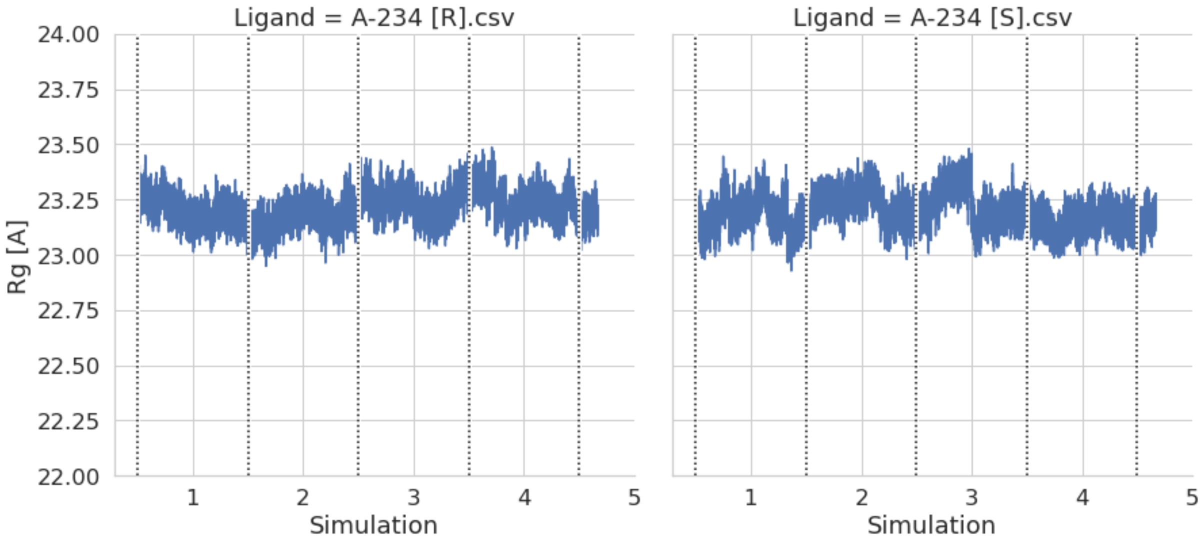

Figure S4. Radius of gyration of protein-A-234 complex as a function of simulation steps

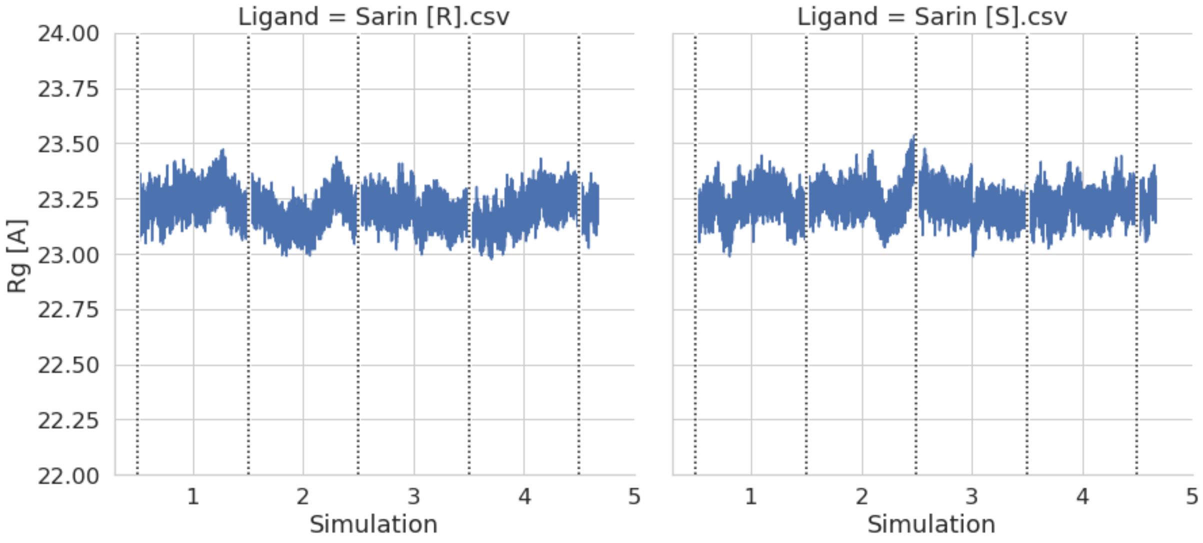

Figure S5. Radius of gyration of protein-sarin complex as a function of simulation steps

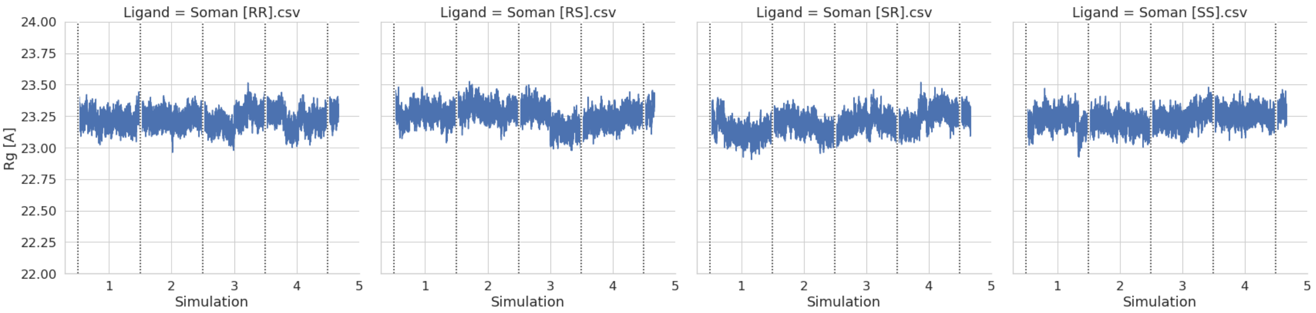

Figure S6. Radius of gyration of protein-soman complex as a function of simulation steps

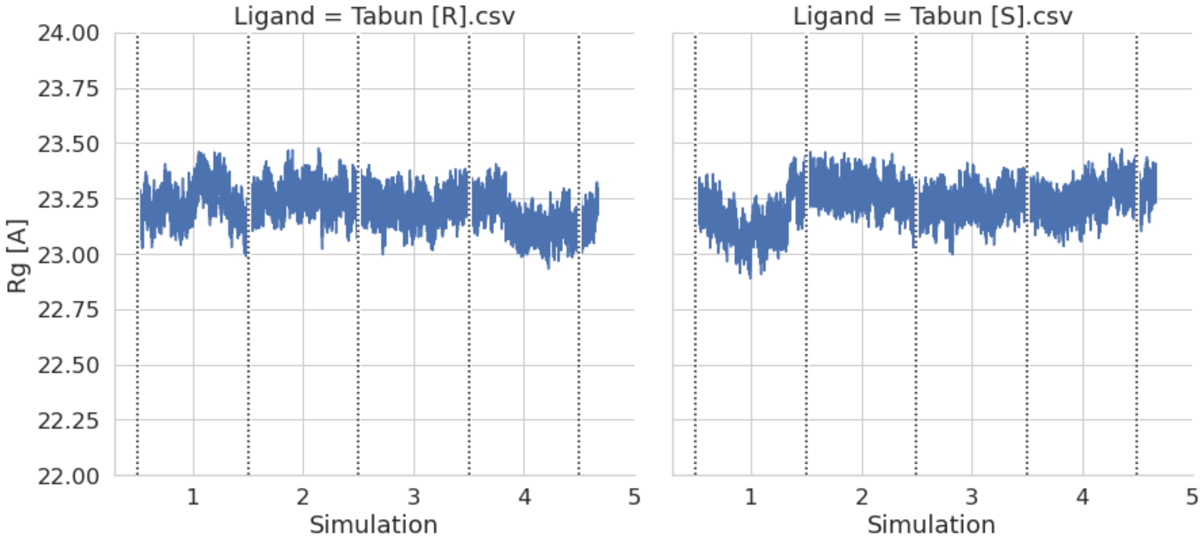

Figure S7. Radius of gyration of protein-tabun complex as a function of simulation steps

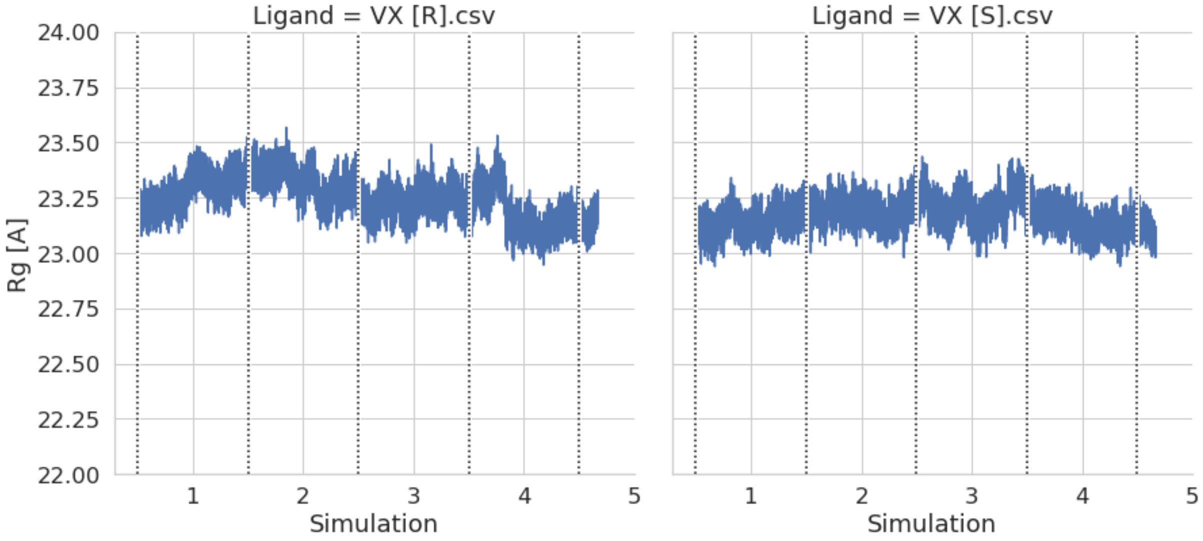

Figure S8. Radius of gyration of protein-VX complex as a function of simulation steps

Figures S9-S16

RMSD of protein-ligand complex as a function of simulation steps

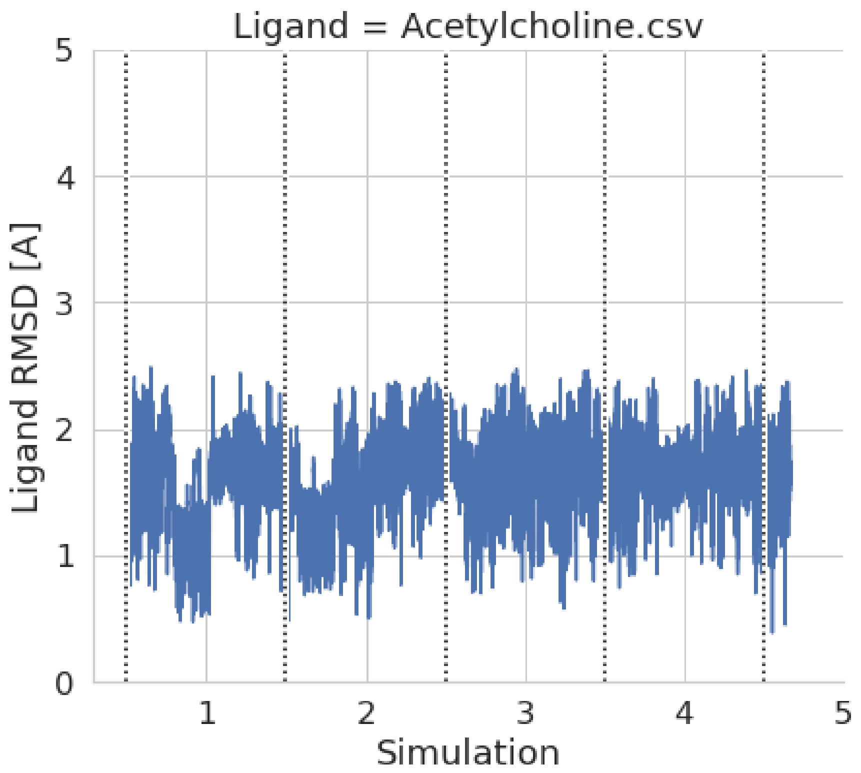

Figure S9. RMSD of protein-acetylcholine complex as a function of simulation steps

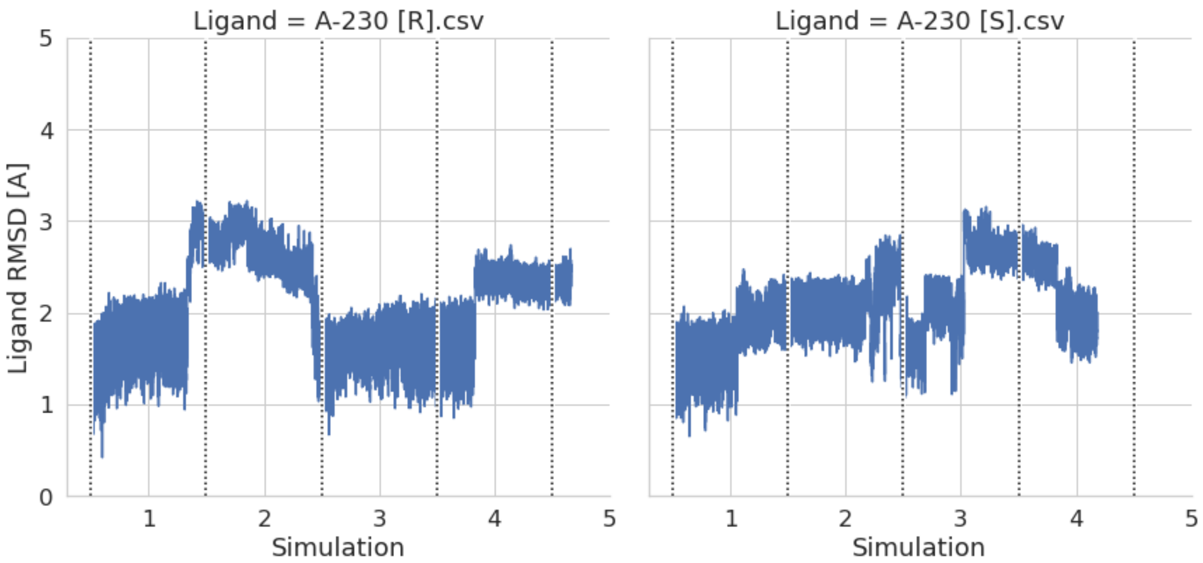

Figure S10. RMSD of protein-A-230 complex as a function of simulation steps

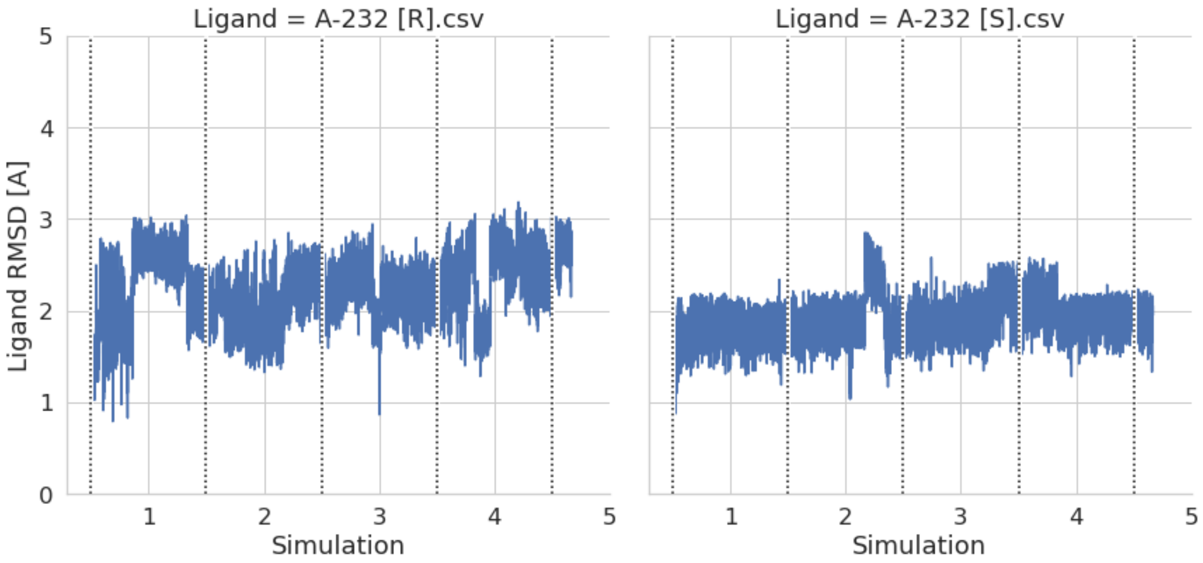

Figure S11. RMSD of protein-A-232 complex as a function of simulation steps

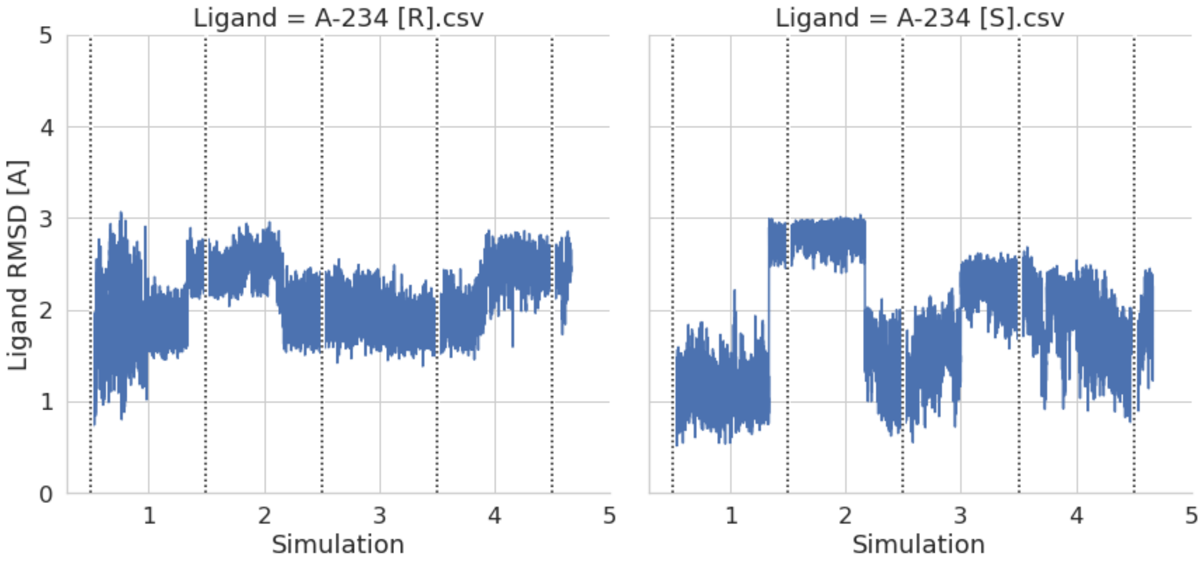

Figure S12. RMSD of protein-A234 complex as a function of simulation steps

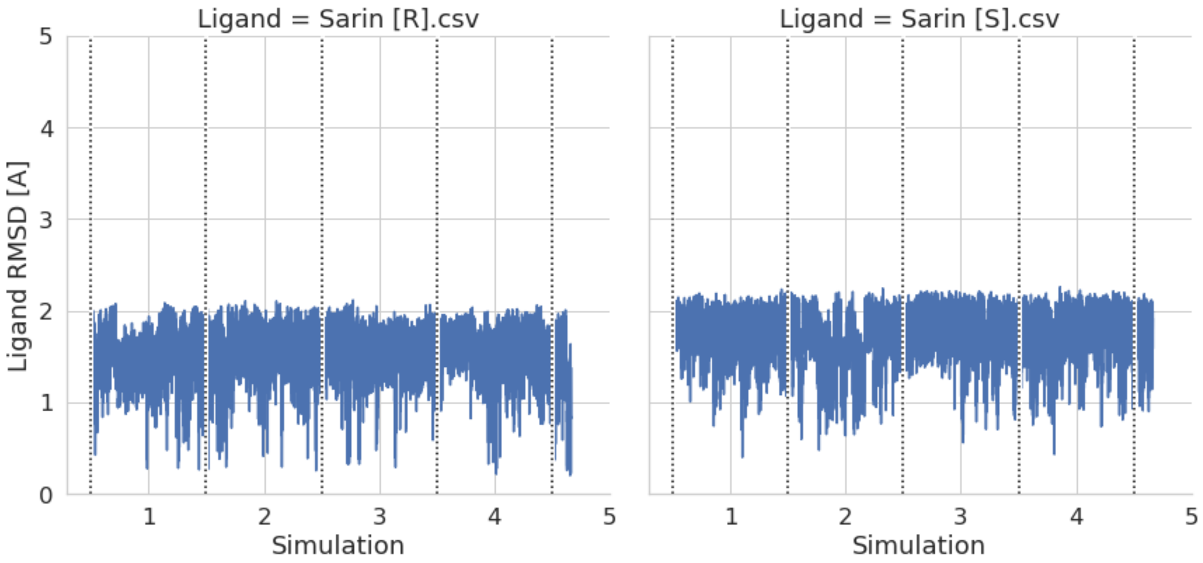

Figure S13. RMSD of protein-sarin complex as a function of simulation steps

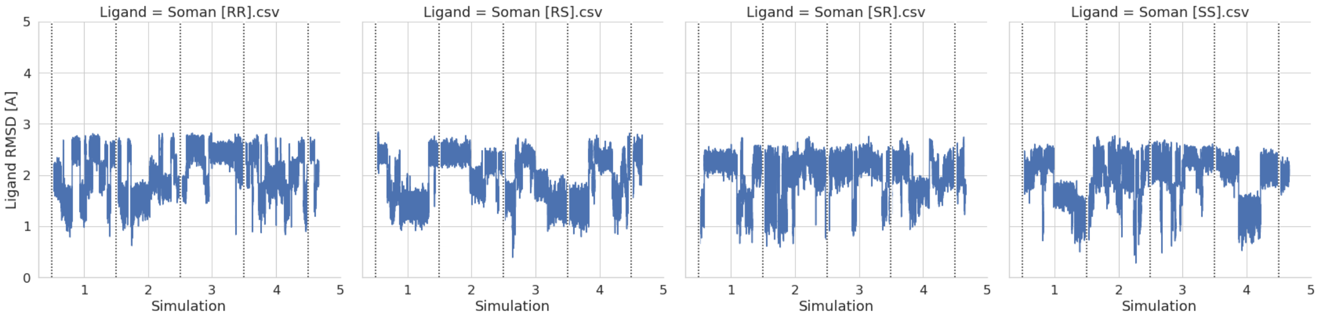

Figure S14. RMSD of protein-soman complex as a function of simulation steps

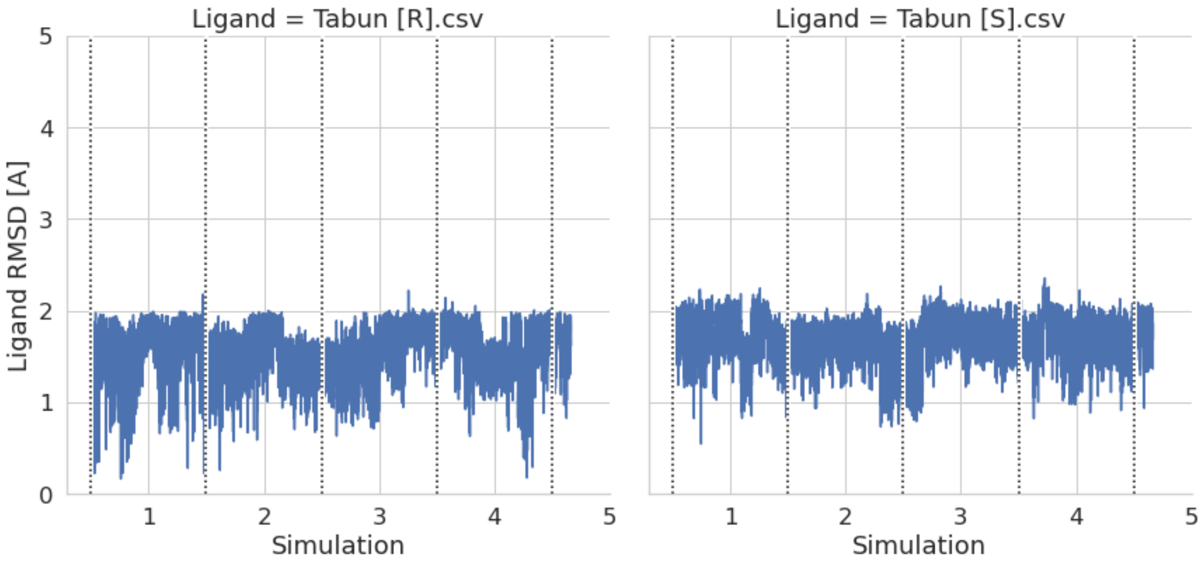

Figure S15. RMSD of protein-tabun complex as a function of simulation steps

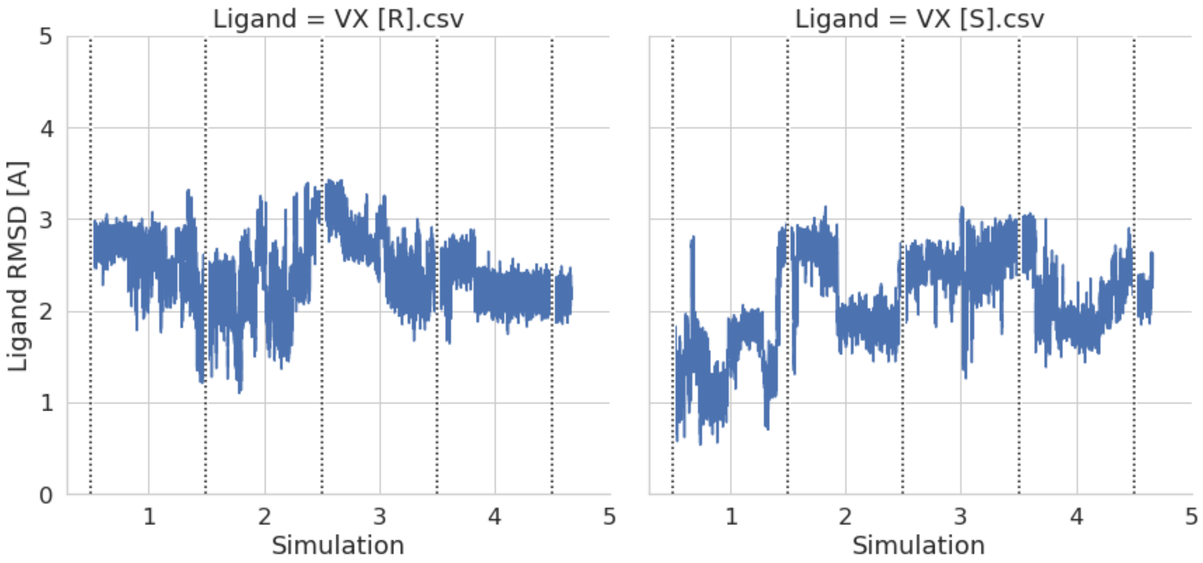

Figure S16. RMSD of protein-VX complex as a function of simulation steps

Figures S17-S24

<u>C_α RMSD of protein-ligand complex as a function of simulation steps</u>

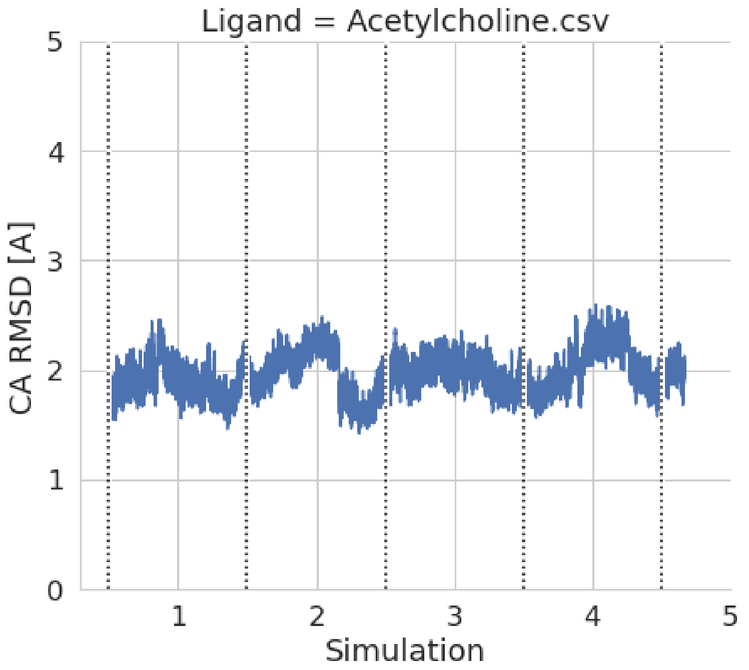

Figure S17. C_α RMSD of protein-acetylcholine complex as a function of simulation steps

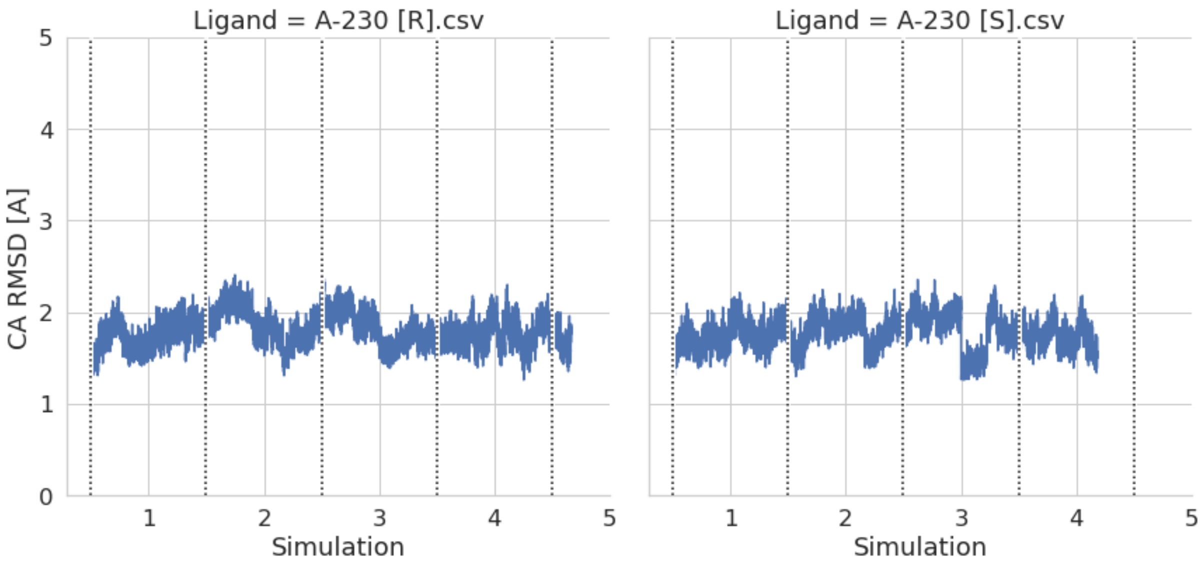

Figure S18. C_α RMSD of protein-A-230 complex as a function of simulation steps

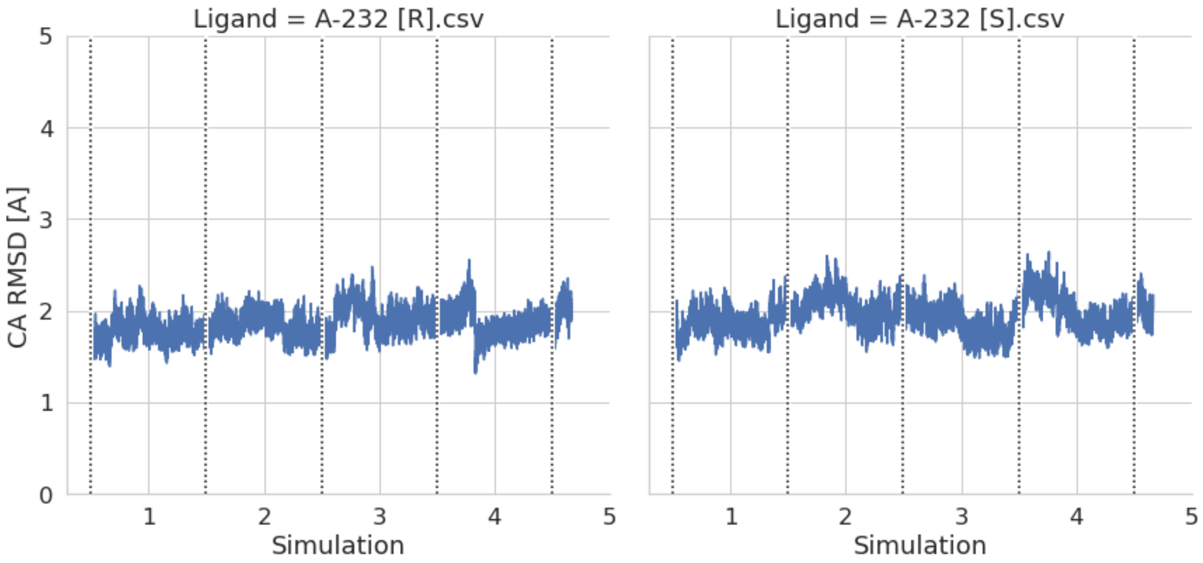

Figure S19. C_α RMSD of protein-A-232 complex as a function of simulation steps

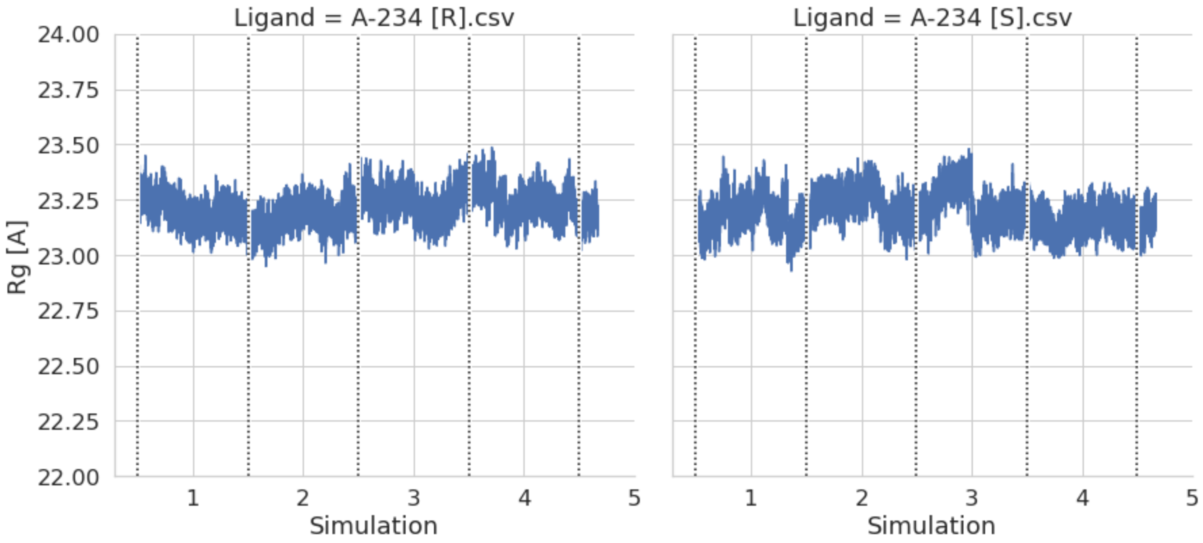

Figure S20. C_α RMSD of protein-A-234 complex as a function of simulation steps

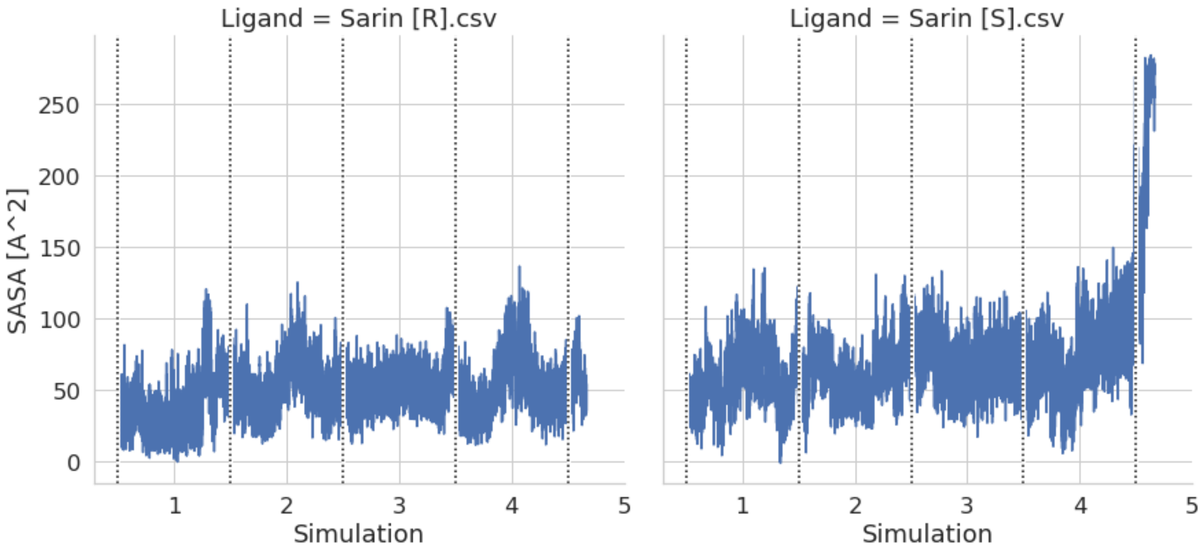

Figure S21. C_α RMSD of protein-sarin complex as a function of simulation steps

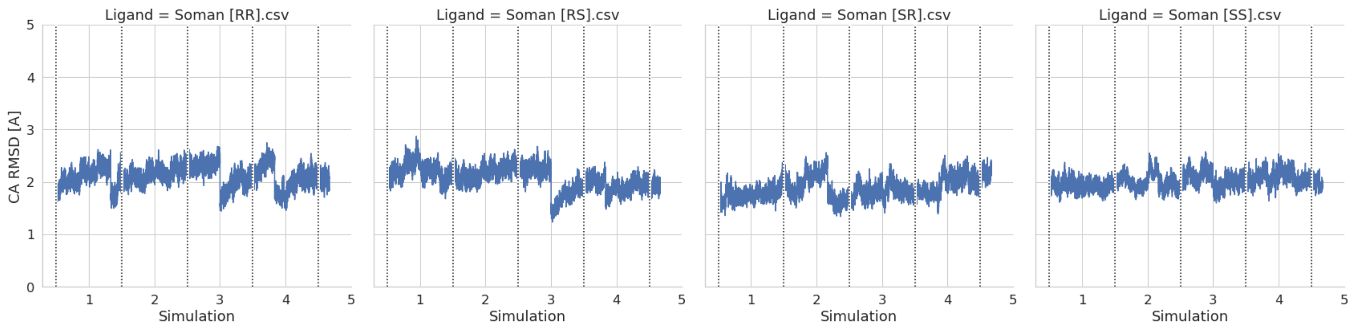

Figure S22. C_α RMSD of protein-soman complex as a function of simulation steps

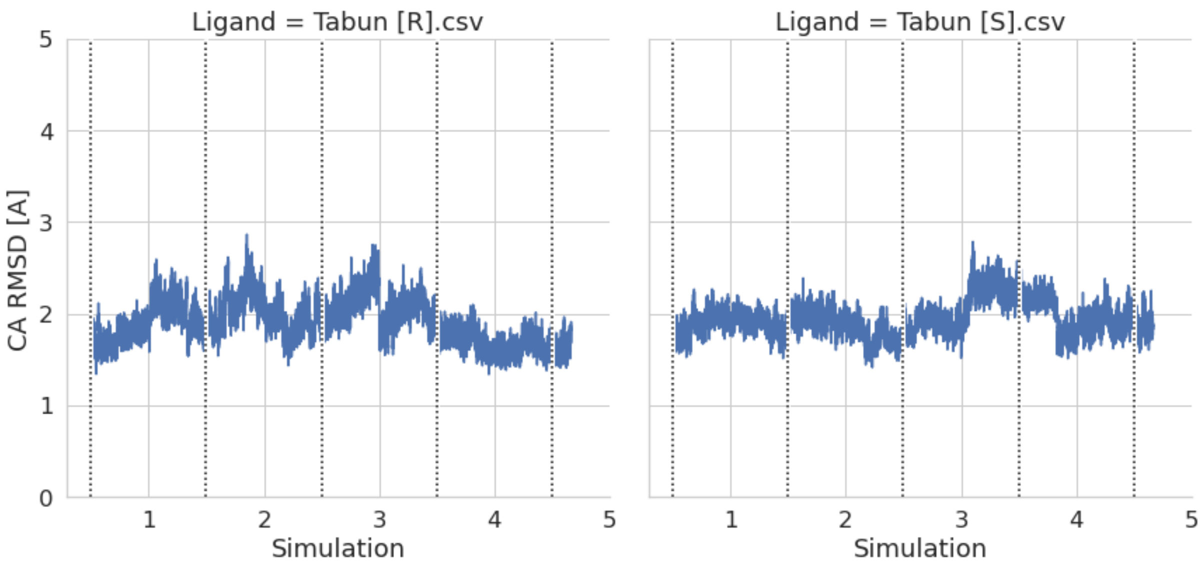

Figure S23. C_α RMSD of protein-tabun complex as a function of simulation steps

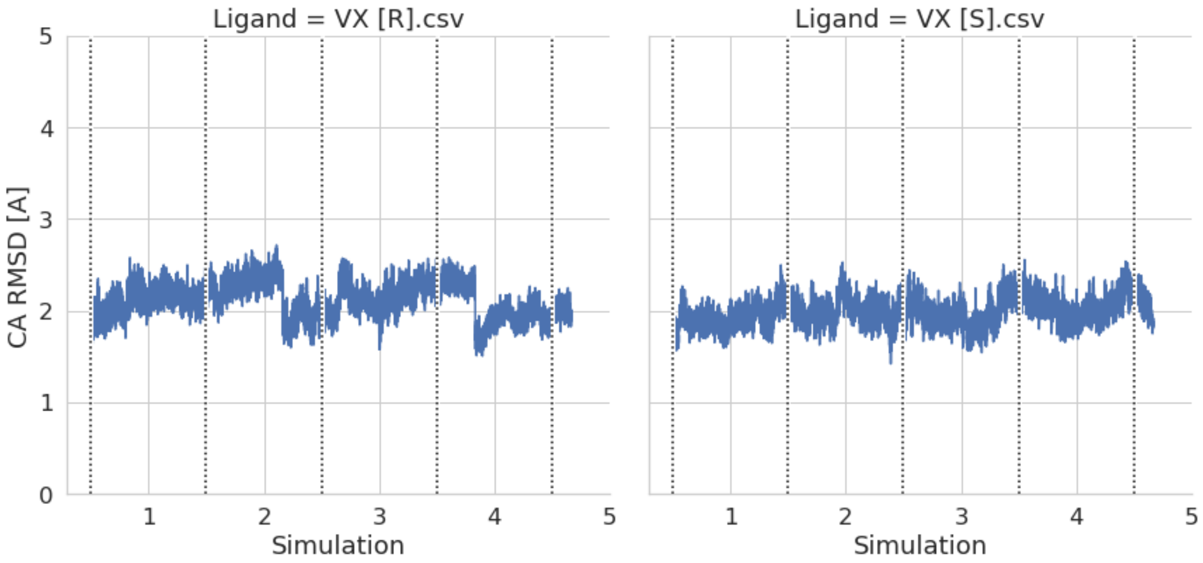

Figure S24. C_α RMSD of protein-VX complex as a function of simulation steps

Figures S25-S32

<u>SASA of protein-ligand complex as a function of simulation steps</u>

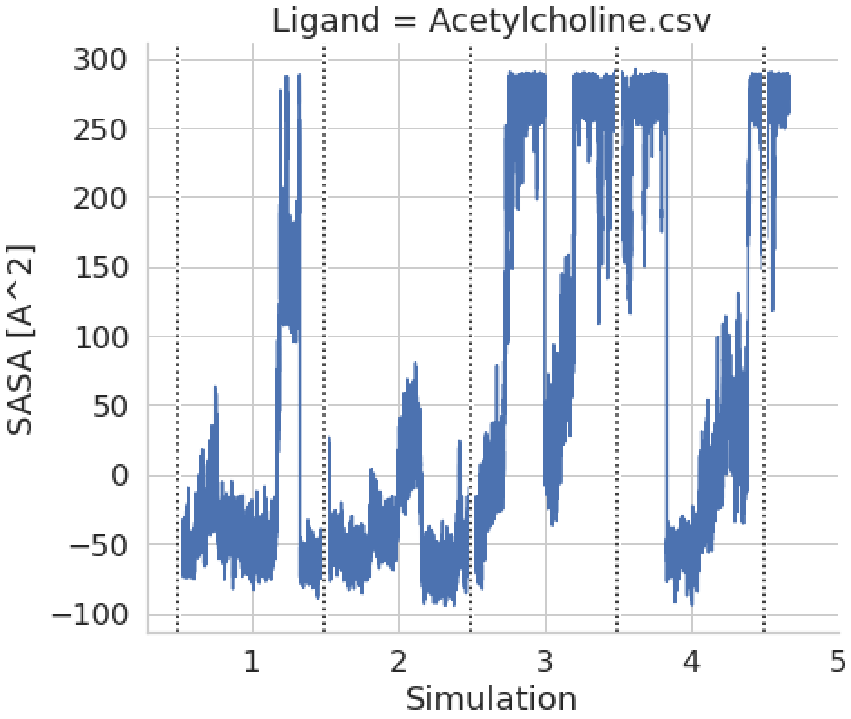

Figure S25. SASA of protein-acetylcholine complex as a function of simulation steps

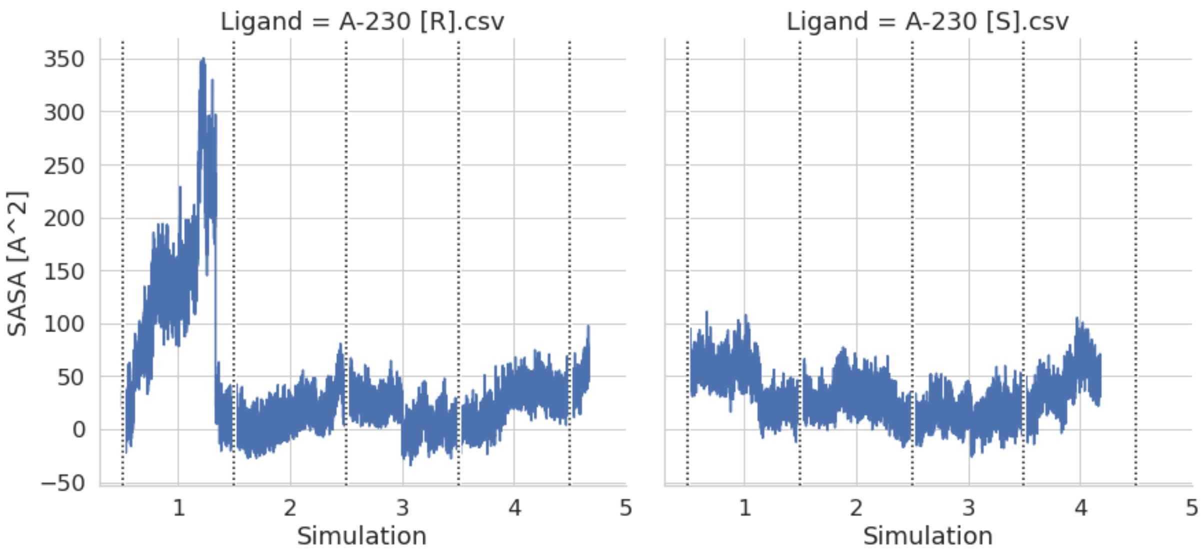

Figure S26. SASA of protein-A-230 complex as a function of simulation steps

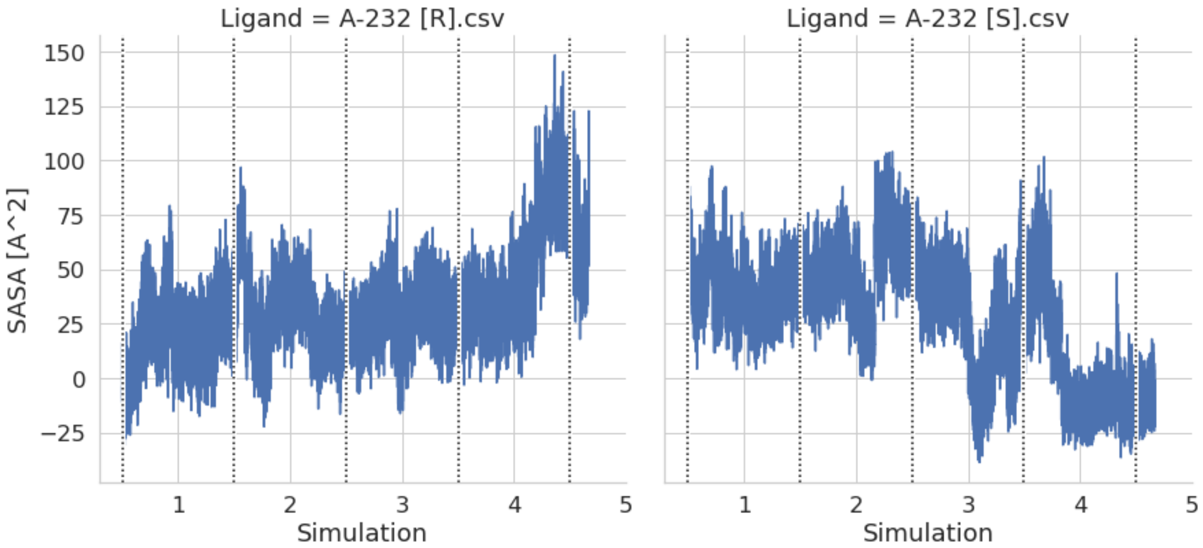

Figure S27. SASA of protein-A-232 complex as a function of simulation steps

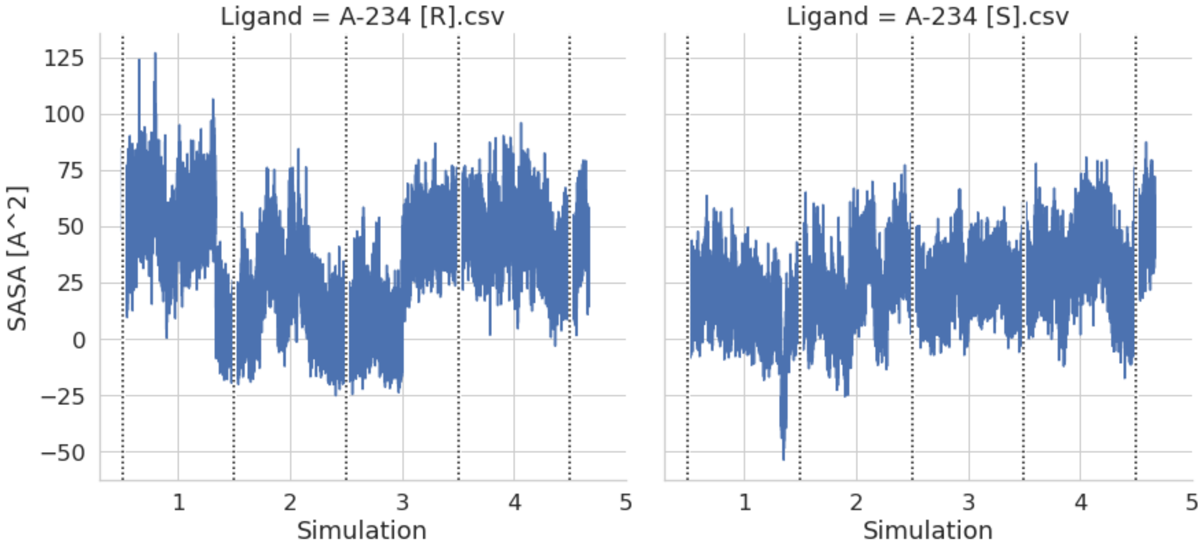

Figure S28. SASA of protein-A-234 complex as a function of simulation steps

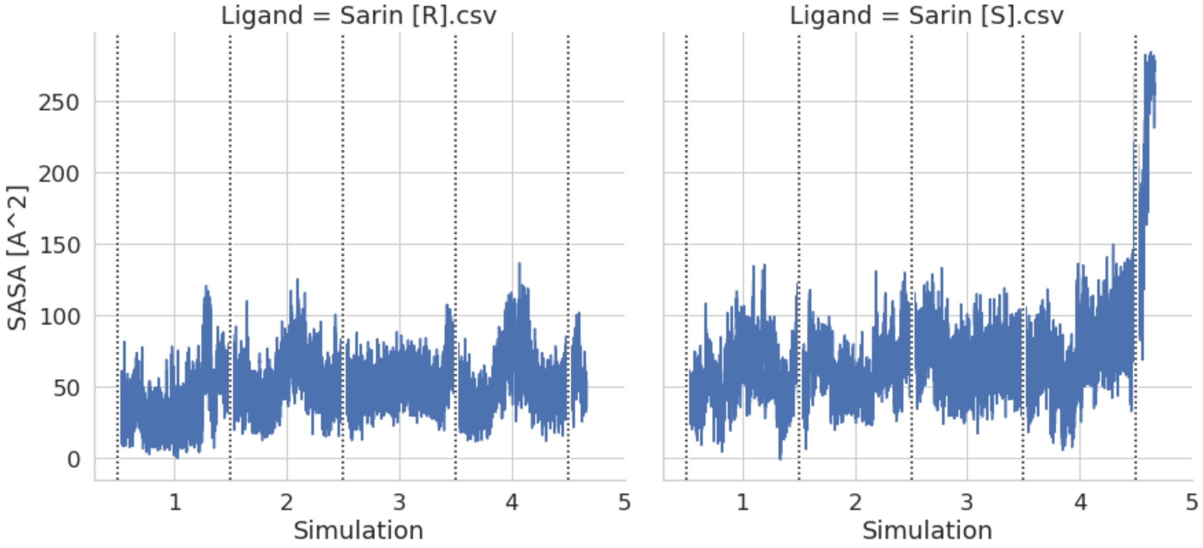

Figure S29. SASA of protein-sarin complex as a function of simulation steps

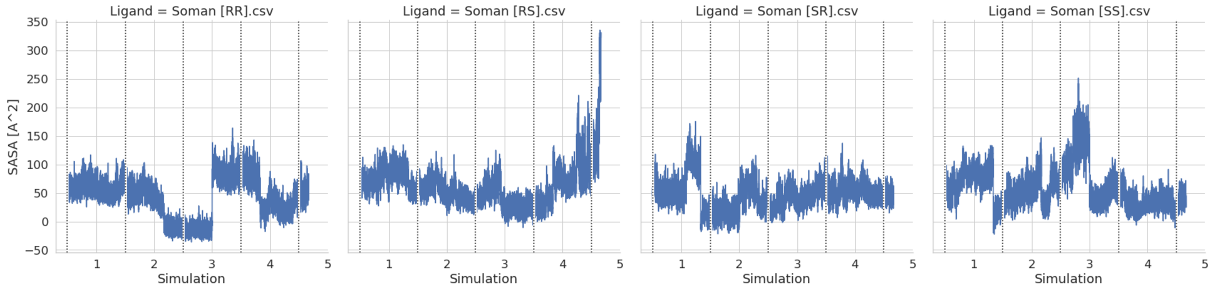

Figure S30. SASA of protein-soman complex as a function of simulation steps

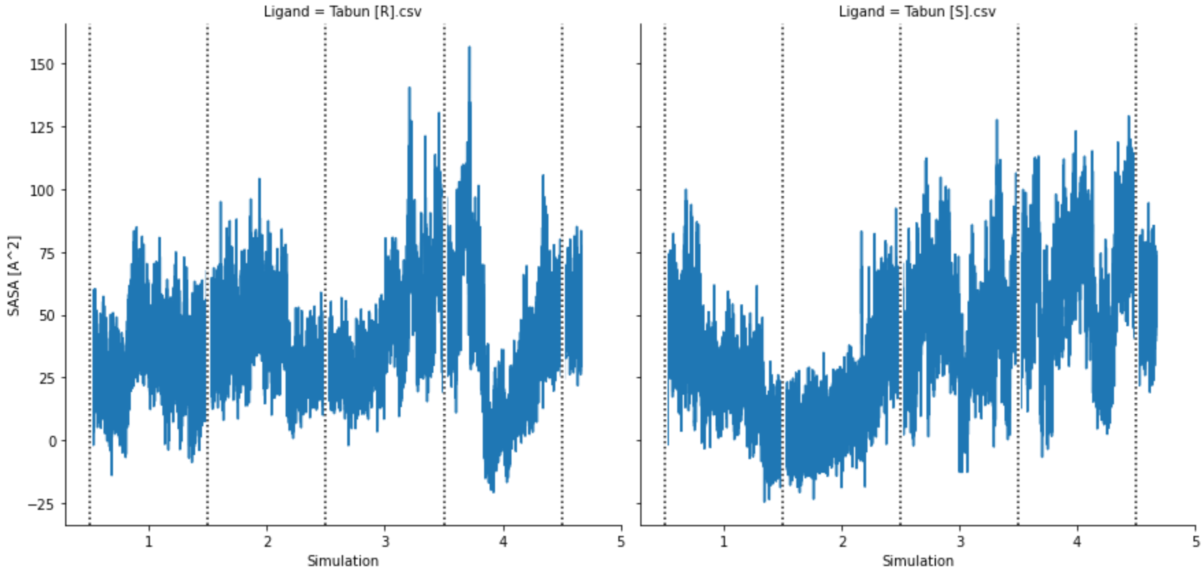

Figure S31. SASA of protein-tabun complex as a function of simulation steps

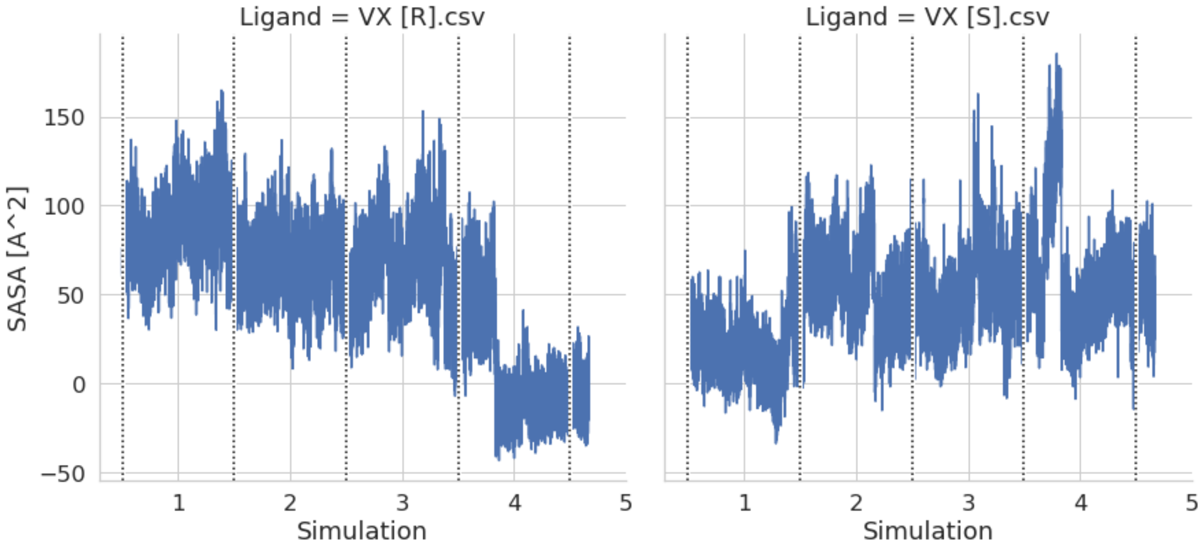

Figure S32. SASA of protein-VX complex as a function of simulation steps

Figures S33-S38

Interaction partitioning for the given ligands
(for the sake of clarity, we intentionally omitted the residue names in the exemplary snapshots)

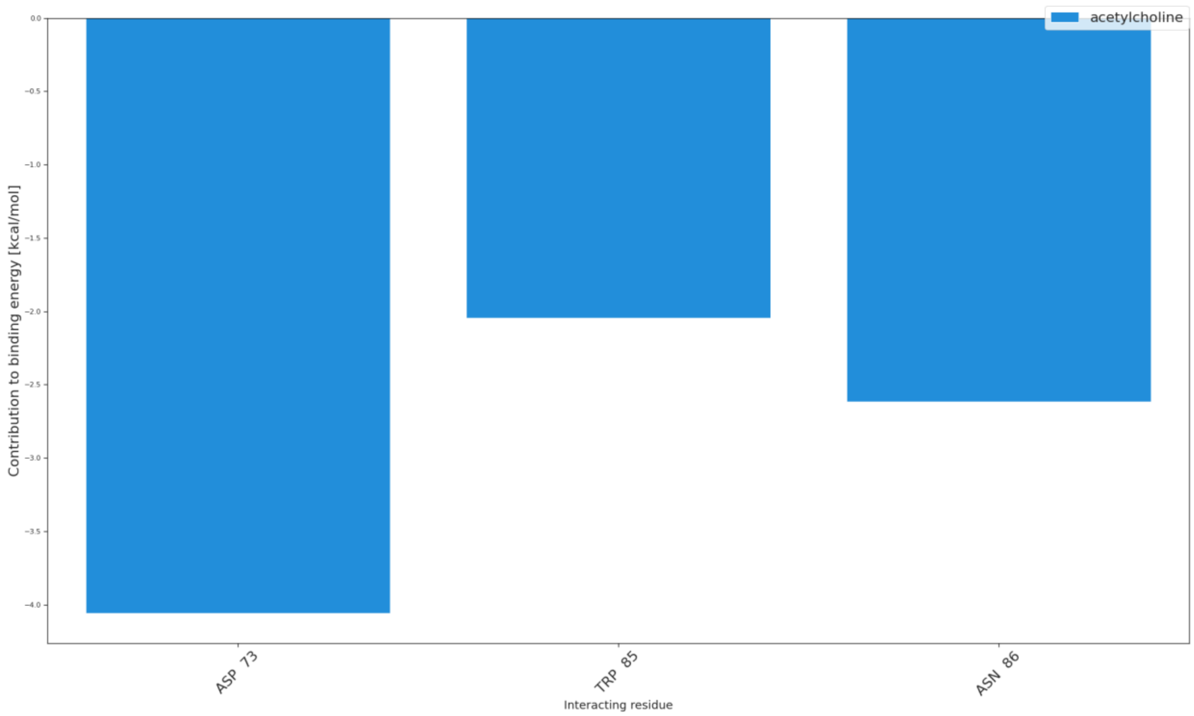

Figure S33. Interaction partitioning of protein-acetylcholine complex

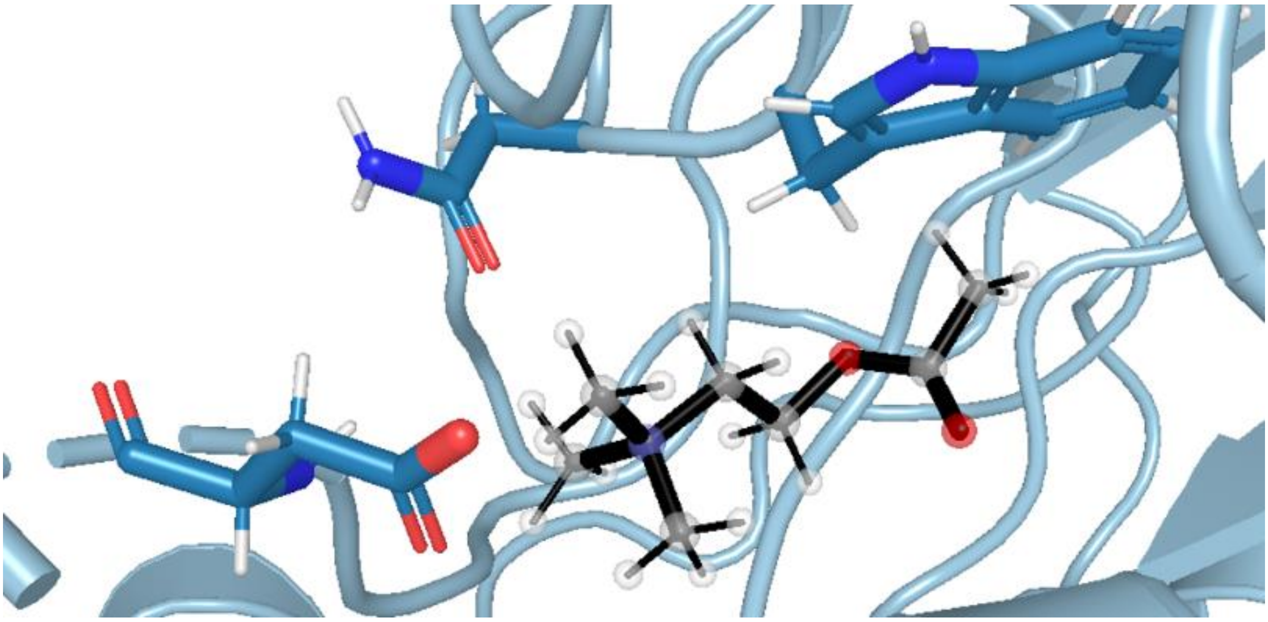

Figure S34. Exemplary snapshot of protein-acetylcholine complex surrounded by all interacting aminoacids specific for the natural agonist

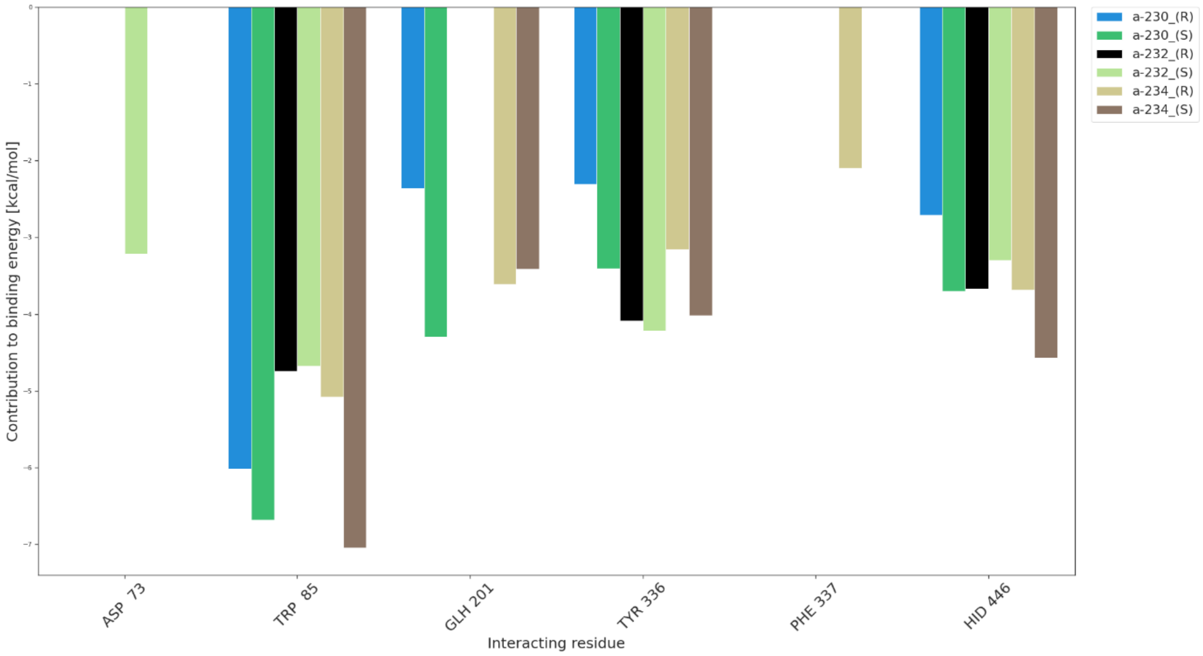

Figure S35. Interaction partitioning of protein-Novichoks complexes

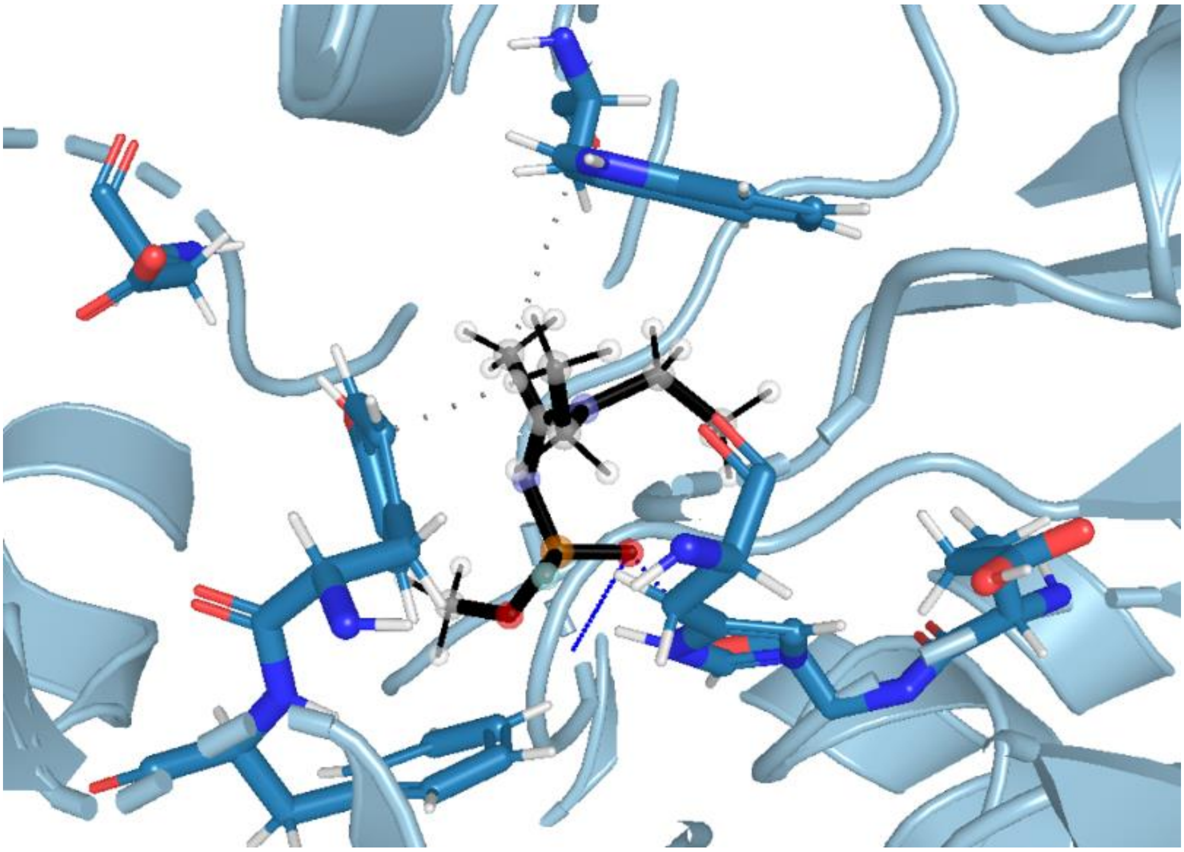

Figure S36. Exemplary snapshot of protein-A-232 complex surrounded by all interacting aminoacids specific for this tier

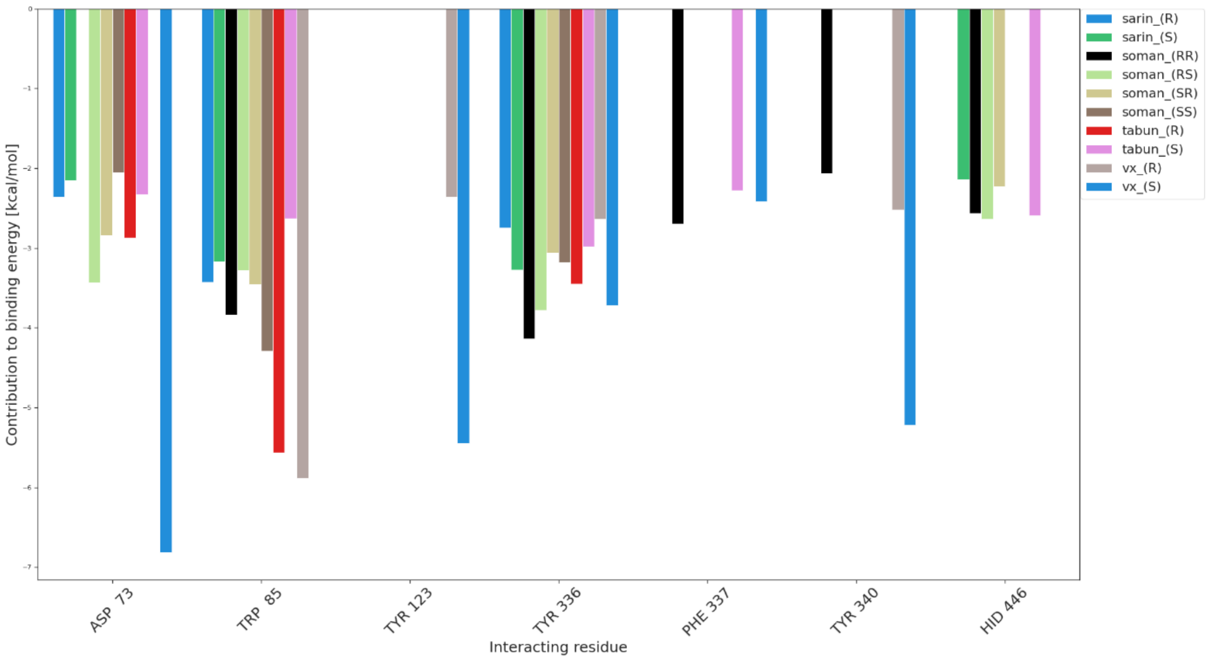

Figure S37. Interaction partitioning of protein-other OPNAs complexes

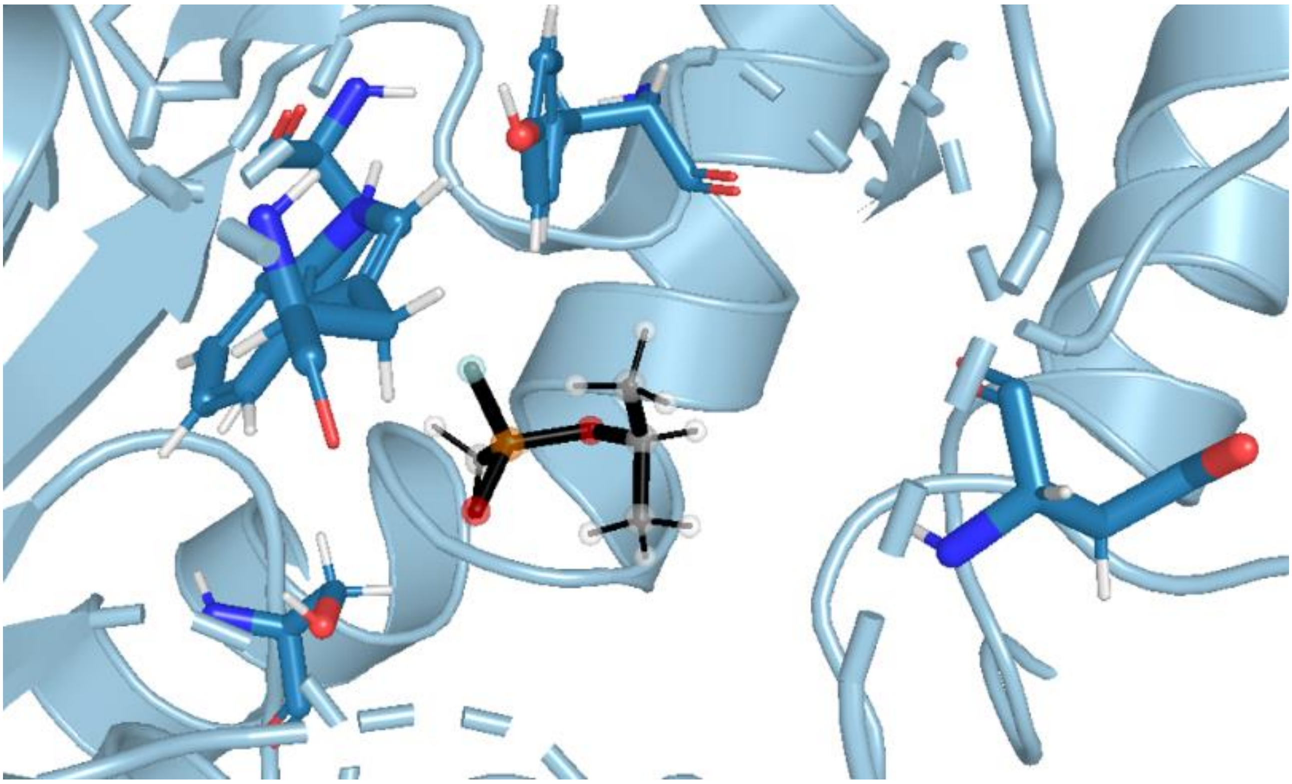

Figure S38. Exemplary snapshot of protein-sarin complex surrounded by all interacting aminoacids specific for this tier
